# Detection of volcanic unrest onset in La Palma, Canary Islands, evolution and implications

**DOI:** 10.1038/s41598-021-82292-3

**Published:** 2021-01-28

**Authors:** José Fernández, Joaquín Escayo, Zhongbo Hu, Antonio G. Camacho, Sergey V. Samsonov, Juan F. Prieto, Kristy F. Tiampo, Mimmo Palano, Jordi J. Mallorquí, Eumenio Ancochea

**Affiliations:** 1 Instituto de Geociencias (CSIC, UCM), Calle del Doctor Severo Ochoa, no 7, Ciudad Universitaria, 28040 Madrid, Spain; 2 CommSensLab-Universitat Politècnica de Catalunya, D3-Campus Nord-UPC, C. Jordi Girona 1-3, 08034 Barcelona, Spain; 3 Canada Centre for Mapping and Earth Observation, Natural Resources Canada, 560 Rochester Street, Ottawa, ON K1A 0E4 Canada; 4 ETS de Ingenieros en Topografía, Geodesia y Cartografía, Universidad Politécnica de Madrid, 28031 Madrid, Spain; 5 Cooperative Institute for Research in Environmental Sciences (CIRES), 216UCB, University of Colorado at Boulder, Boulder, CO 80309 USA; 6 Istituto Nazionale di Geofisica e Vulcanologia, Osservatorio Etneo - Sezione di Catania, Piazza Roma 2, 95125 Catania, Italy; 7 Departamento de Mineralogía y Petrología, Fac. CC. Geológicas, Universidad Complutense de Madrid, 28040 Madrid, Spain

**Keywords:** Natural hazards, Solid Earth sciences

## Abstract

La Palma island is one of the highest potential risks in the volcanic archipelago of the Canaries and therefore it is important to carry out an in-depth study to define its state of unrest. This has been accomplished through the use of satellite radar observations and an original state-of-the-art interpretation technique. Here we show the detection of the onset of volcanic unrest on La Palma island, most likely decades before a potential eruption. We study its current evolution seeing the spatial and temporal changing nature of activity at this potentially dangerous volcano at unprecedented spatial resolutions and long time scales, providing insights into the dynamic nature of the associated volcanic hazard. The geodetic techniques employed here allow tracking of the fluid migration induced by magma injection at depth and identifying the existence of dislocation sources below Cumbre Vieja volcano which could be associated with a future flank failure. Therefore they should continue being monitored using these and other techniques. The results have implications for the monitoring of steep-sided volcanoes at oceanic islands.

## Introduction

Over the last two decades, episodes of volcanic unrest have been observed in the Canaries. Examples include the anomalous seismicity detected on Tenerife in 2004–2005, the recent eruption in El Hierro in 2011–2012, and the anomalous seismicity in La Palma in 2017 and 2018^1–4^. Considering the important risk to the economy and population in light of this activity, there is a compelling case for an in-depth study to define the state of unrest on the different islands in the archipelago.

**Figure 1:**
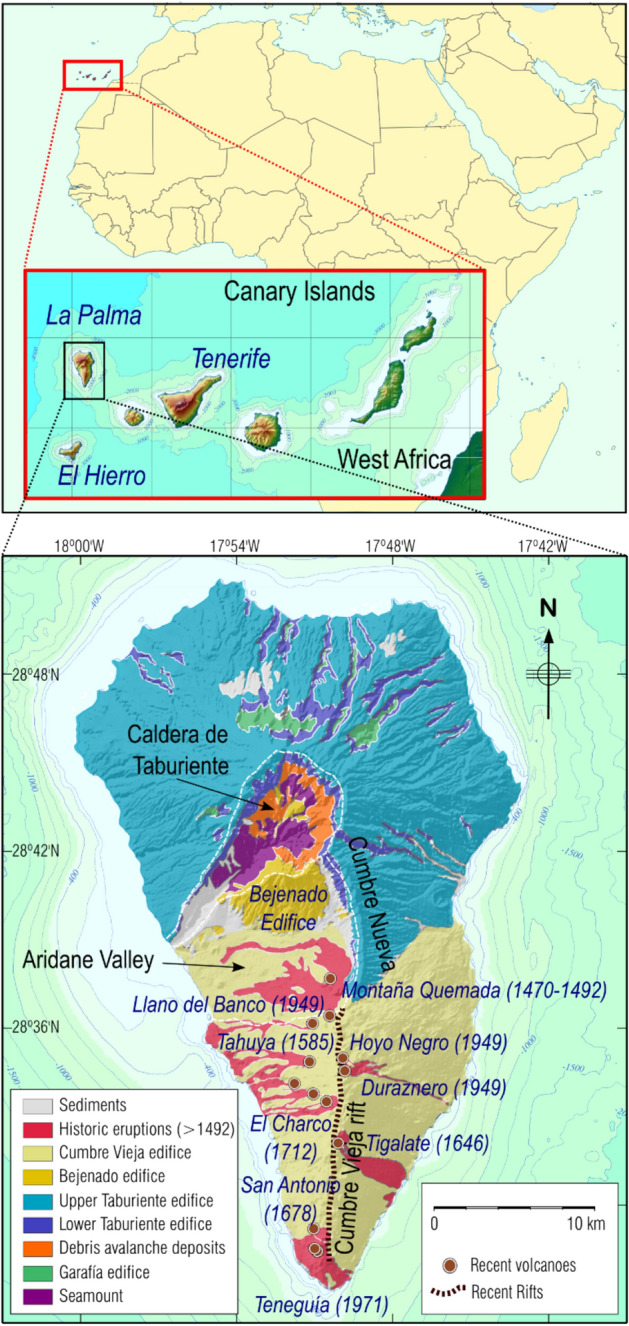
Geographical location and geological elements. Location of Canary Islands and La Palma Island (top panel) and simplified geological map (lower panel). See insets for scale and key. GMT software (www.generic-maping-tools.org) was used to create this figure.

The westernmost and youngest islands of the Canary archipelago are El Hierro and La Palma (Fig. 1). La Palma has been volcanically active for at least 4 Ma. It is made up of two main volcanic units: a large volcanic complex in the north (northern volcanic complex, NVC), with an older, circular morphology, and a younger volcanic rift developed along the southern portion of the island, the Cumbre Vieja (CV) edifice.

The NVC is the result of the superposition of several basaltic volcanic edifices. The older edifice consists of a submarine volcanic body aged between 3 and 4 Ma^5^, which was built from a depth below sea level (bsl) of 4000 m. Superimposed on the older edifice, three large subaerial volcanic structures developed from 1.7 Ma to 0.4 Ma^6,7^, the Garafía edifice (1.7–1.2 Ma); the Taburiente-Cumbre Nueva edifice, T-CN, (1.2–0.4 Ma) and the Bejenado edifice (0.56–0.49 Ma) (Fig. 1). Currently, the NVC reaches 2426 m altitude. Great landslides and intense erosion have affected the NVC, exposing both the submarine structure and the plutonic bodies and dikes that constitute the roots of the edifices at the bottom of the Caldera de Taburiente^8^. The submarine part of the island is inclined and raised to about 300 m altitude^9^, under the Garafía and Taburiente edifices, and now can be seen at the bottom of the caldera. The T-CN edifice, which covers the older ones, its first phase (1.2–0.8 Ma) morphology had broad circular features, providing the current shape in the north of the island. However, in a second phase (0.8–0.4 Ma), its morphology is the same as the approximately N-S Cumbre Nueva (CN) rift.

The second large volcanic unit is the CV edifice. Although it is not known when the activity began, it was later than the previous edifices. The rift is oriented N-S, as in the case of the CN, with a maximum altitude of 1950 m, a subaerial length of approximately 17 km which extends several kilometers on the seabed^10^. The most recent volcanic activity on the island is concentrated in this volcanic edifice, including the six historical eruptions that have taken place in the last 500 years^11^ (Fig. 1). One of the last events was the San Juan Eruption in 1949 when eruptive activity occurred at three vents: Duraznero, Llano del Banco and Hoyo Negro. The last sub-aerial eruption in La Palma and the Canaries was the 1971 Teneguía eruption, approximately 50 years ago (Fig. 1). Volcanic unrest in La Palma, and especially in the CV area, also has been associated with landslide and tsunami hazards^12^. Additionally, Torres-González et al.^4^ found evidence of a stalled magmatic intrusion at about 25 km depth in La Palma, based on seismic and geochemical anomalies during 2017–2018. The fact that they did not detect surface deformation for that event, combined with the previously described hazards, motivated the study of the surface deformation on La Palma using differential interferometric synthetic aperture radar (DInSAR).

Historically, seismicity has been the first precursor detected in unrest processes in the Canaries^1–4,13^, while the surface deformation measured during unrest episodes have not been large. Deformation of around ten cm was measured a few months before the eruption at El Hierro. On Tenerife, during its 2004–2005 unrest, the detected deformation was on the order of 2–3 cm, sometimes with a low signal/noise ratio^1^. Measuring displacements using DInSAR, the situation is further complicated by the existence of steep-sided volcanoes where atmospheric effects are significant^1,13–15^, as atmospheric effects and deformation signal coincide, making it more difficult to retrieve the deformation. Using GNSS campaigns, the small magnitude of the displacement and the limited number of available observation sites^16,17^ makes it difficult to detect and interpret the displacements^18^. Additionally, on La Palma, as in many volcanic islands, the location of possible unrest-related deformation is not known a priori and may vary spatially in time^17^. This means that sparse GNSS networks may not be useful for detecting small magnitude deformation, primarily in the early stages of unrest^4,17^.

Here we overcome these limitations by using advanced DInSAR techniques, including the correction of atmospheric artifacts, facilitating the availability of a large number of high precision data. They are combined with a state-of-the-art inversion technique^19^ that carries out a multi-source inversion of ground deformation data using a nonlinear approach which permits the determination of location, size and three-dimensional configuration without any a priori assumption on the number, nature or shape of the causative sources. The results are discussed considering the 3D cortical structure of the Island determined from gravimetric observations^20,21^.

## Results

### DInSAR results

The DInSAR study was carried out using Single Look Complex (SLC) images from the European Space Agency’s (ESA) ENVISAT (2006–2010) and Sentinel-1 (2017–2020) C-band satellites. Both ascending and descending acquisition geometries were processed for the two satellites. Additionally, descending images were acquired by the Canadian Space Agency (CSA) C-band RADARSAT-2 satellite for the period 2010–2017. The topographic phase was removed using a high-quality Digital Terrain Model derived from the Instituto Geográfico Nacional (IGN)^22^.

To compute line of sight (LOS) surface displacements, the Coherent Pixel Technique (CPT) was employed^23^ for processing ENVISAT and Sentinel-1 data sets. An SBAS approach with a multilook window of 5 × 25 samples was applied with coherence criteria of 0.4 to evaluate the pixel’s phase quality. To remove the atmospheric phase screen (APS), due to the strong topographic variation characterizing the island, we corrected the ENVISAT interferograms with a spatio-temporal filtering approach and the Sentinel-1 by using the latest ERA5 global reanalysis data^14^. An example of the APS correction for a Sentinel-1 interferogram is given in Supplementary Fig. 1. The RADARSAT-2 dataset was processed using MSBAS software^24^. A 4 × 8 multilook was applied and geocoded products were computed with an approximate resolution of 40 m. An atmospheric correction based on a regression of phase vs elevation is carried out for these data. The “Methods” section provides a detailed description of the interferometric processing.

**Figure 2:**
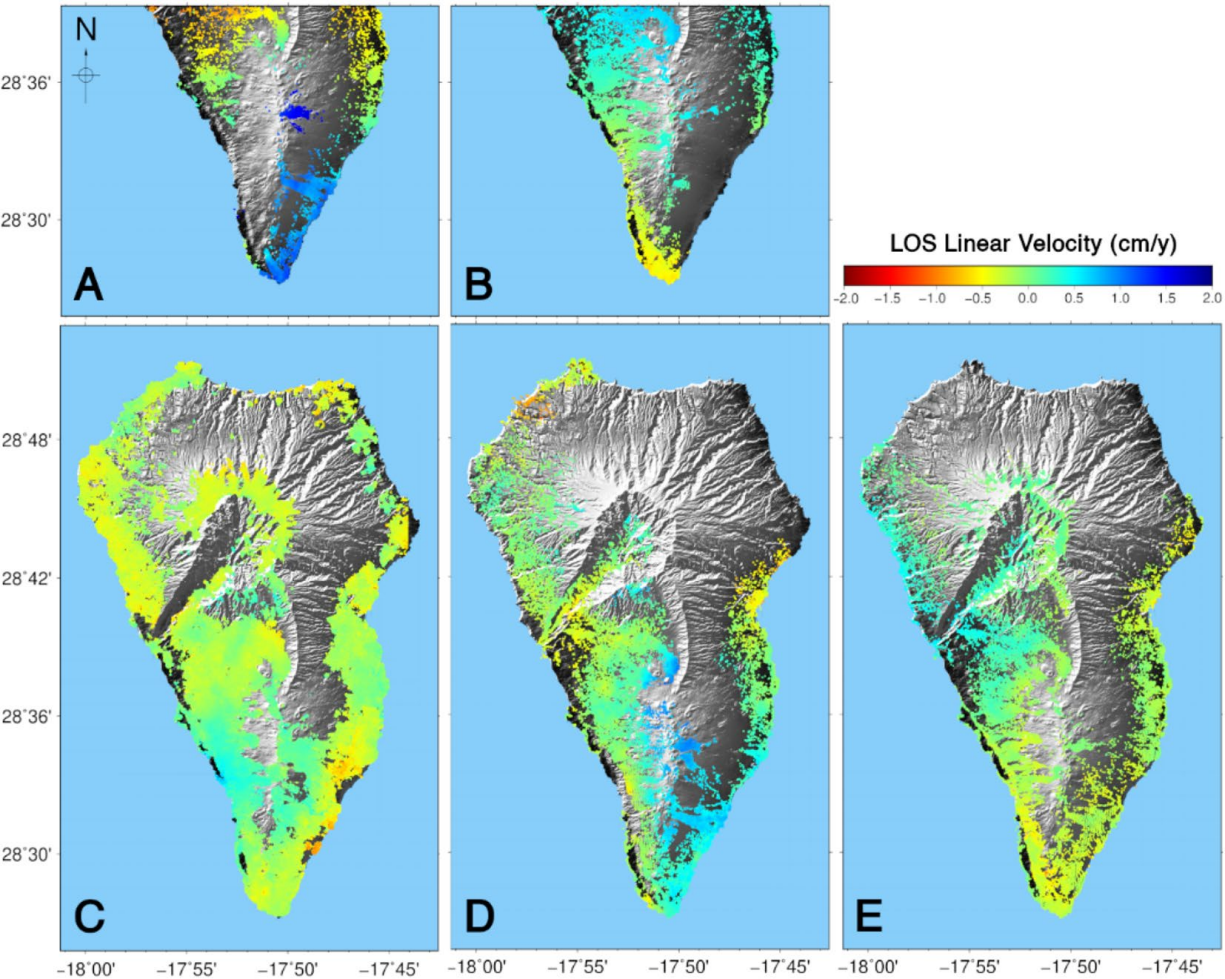
Annual mean LOS velocities obtained for the La Palma Island. (**A**) For the period 2006–2010 results obtained with SAR images from the ENVISAT satellite acquired in ascending orbits; (**B**) same for images acquired in descending orbits. (**C**) Results obtained using images from RADARSAT-2 satellite acquired in descending orbits for the period 2010–2017. Results for 2017–2020, using Sentinel-1 images: (**D**) acquired in ascending orbits; and (**E**) in descending orbits. GMT software (www.generic-maping-tools.org) was used to create this figure.

Mean LOS velocities and displacement time-series were obtained for each satellite geometry and for 2006–2010, 2017–2020 time periods. Only descending results were evaluated for 2010–2017. The resulting LOS mean velocities are shown in Fig. 2. LOS displacement time-series for selected pixels are shown in Supplementary Fig. 2 compared with the projected GNSS displacements for ENVISAT and Sentinel-1 constellation. We observed a quite good adjustment between these two independent techniques validating our DInSAR results. RADARSAT-2 displacements time-series (2010–2017) were not used because only one orbit was available and it was not sufficient for high confidence modeling.

### Inversion results

The LOS deformation data for the three time periods were modeled to estimate the nature, location and geometry of the causative sources using the inversion methodology noted above^19^ (see “Methods” section). The result of the free adjustment of available LOS data, for each period considered, provides the deformation sources as 3D cell aggregations for which the inversion adjustment process automatically assigns a nature or source type (e.g., pressure change, strike-slip, dip-slip or tensile dislocation), magnitude values (MPa for pressure changes and cm for dislocations) and their position and orientation (angles of the dislocation planes).

**Figure 3:**
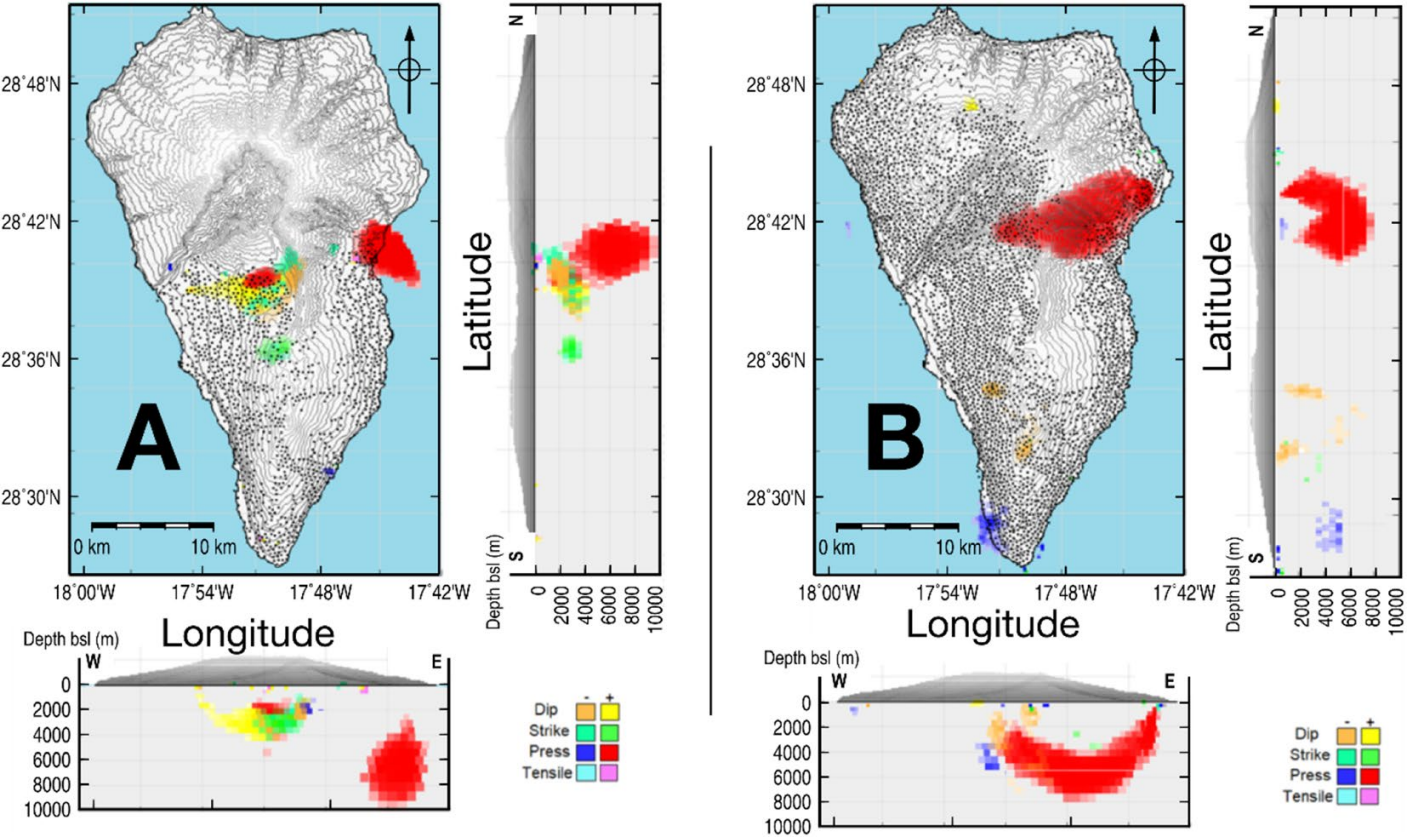
Adjusted model for deformation sources. (**A**) Sources adjusted for the period 2006–2010, and (**B**) for the period 2017–2020. Each time period panel includes planar, E-W vertical and N-S vertical views of significant sources. See color code for the type of source and magnitude. Dots denote the location of ascending and descending LOS deformation data. Matlab (www.mathworks.com) and GMT (www.generic-mapin-tools.org) softwares were used to create this figure.

Modeled sources are plotted in Fig. 3 for the 2006–2010 and 2017–2020 periods, colored according to their nature. Since the spatial coverage of DInSAR results is reduced by decorrelation, we performed a checker-board resolution test to ensure that DInSAR results are sufficient for inversion. We computed ascending and descending LOS for the modeled sources using the models for each kind of source described in the “Methods” section ^19^, and compare the obtained values with the observed ones, Supplementary Figs. 3, 4. The data fit is quite good for both data sets and final residuals for LOS (observed-modeled) have root-mean-squared values lower than 0.4 cm/year.

**Figure 4:**
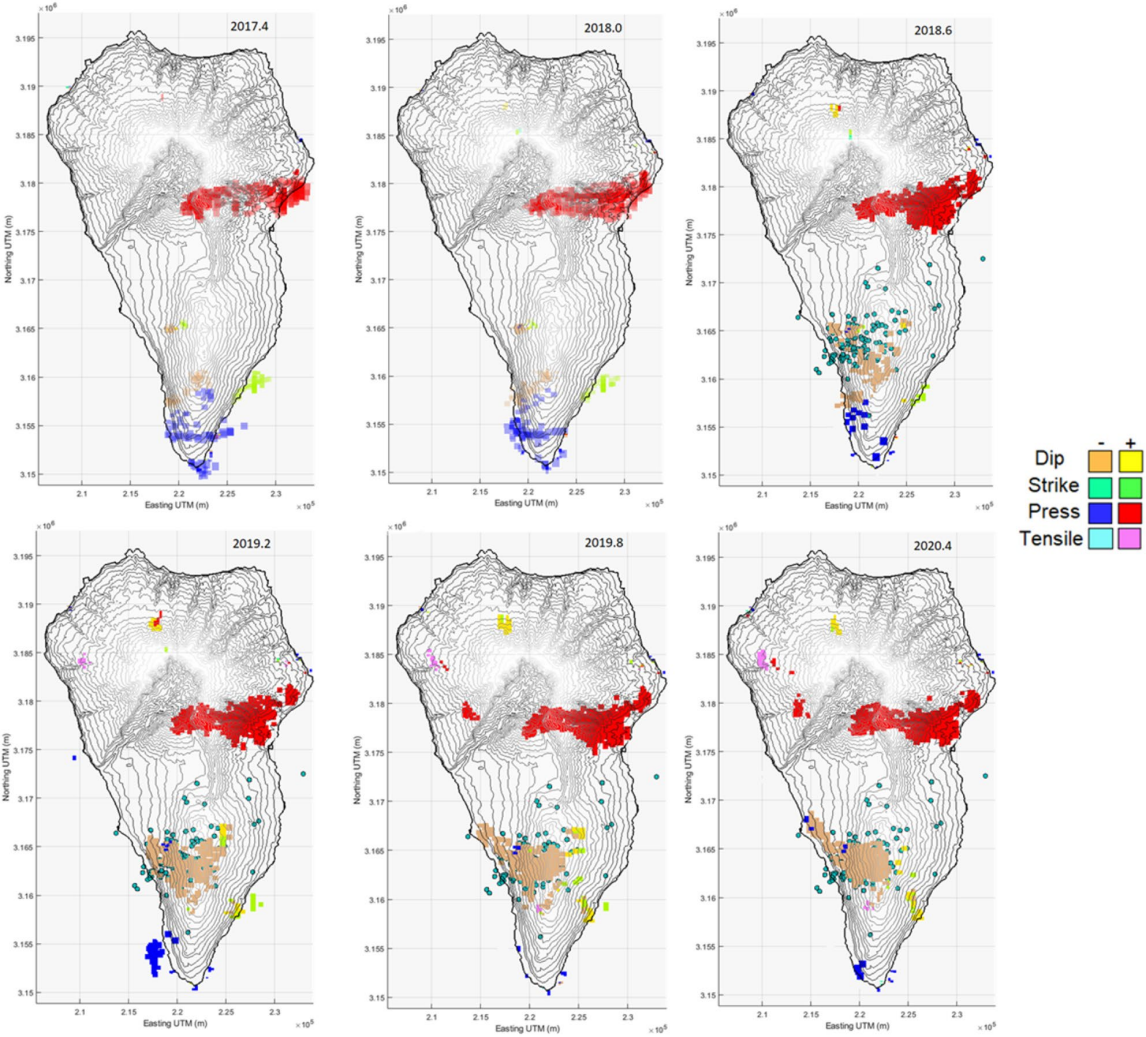
Time evolution of the modeled sources for the 2017–2020 period. The different plots show the time evolution of the different sources obtained inverting the Sentinel-1 ascending and descending LOS displacement time series every 0.6 years. Green circles show the horizontal location of the earthquakes happened during the two seismic swarms (October 2017, February 2018). See Fig. 5 for numbers of seismic events and Fig. 8 for depths of both, events and sources. Matlab software (www.mathworks.com) was used to create this figure.

For the 2010–2017 period, we only have LOS mean velocities obtained from descending data and the atmospheric correction done looks not very reliable in this region probably because of the predominant wind direction that keeps moisture on one side of the island. Inversion results are strongly affected by both of these aspects, see Supplementary Fig. 5. As a result, they are not included in our study.

**Figure 5:**
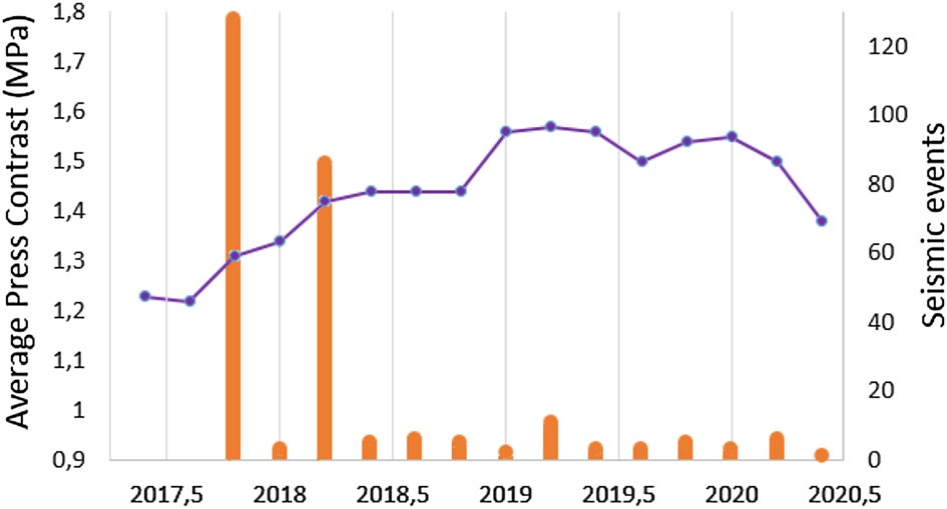
2017–2020 average pressure time evolution for the adjusted sources. Adjusted average pressure per volume unit (blue color) and the number of seismic events (orange columns) between February 2017 and June 2020. During the October 2017 seismic swarm 122 events took place with a magnitude lower than 3 and the 95% of them with magnitude between 1 and 2. During the February 2018 seismic swarm 79 events took place with magnitude lower than 3 and the 77% of them with magnitude between 1 and 2. This figure was created using Microsoft Excel 2016.

For the 2017–2020 period we have been able to study the time evolution of the modeled sources, Fig. 4. The temporal evolution of the positive pressure sources for the period 2017–2020 is shown in Fig. 5. This analysis was not possible for the period 2006–2010 due to the relatively low number and poor temporal distribution of the images.

### Structural gravimetry results

Basic information to discuss the results obtained from the inversion of deformation data is the knowledge of the crustal structure of the island. We use the 3D density distribution below the surface of the island obtained from gravimetric measurements.

**Figure 6:**
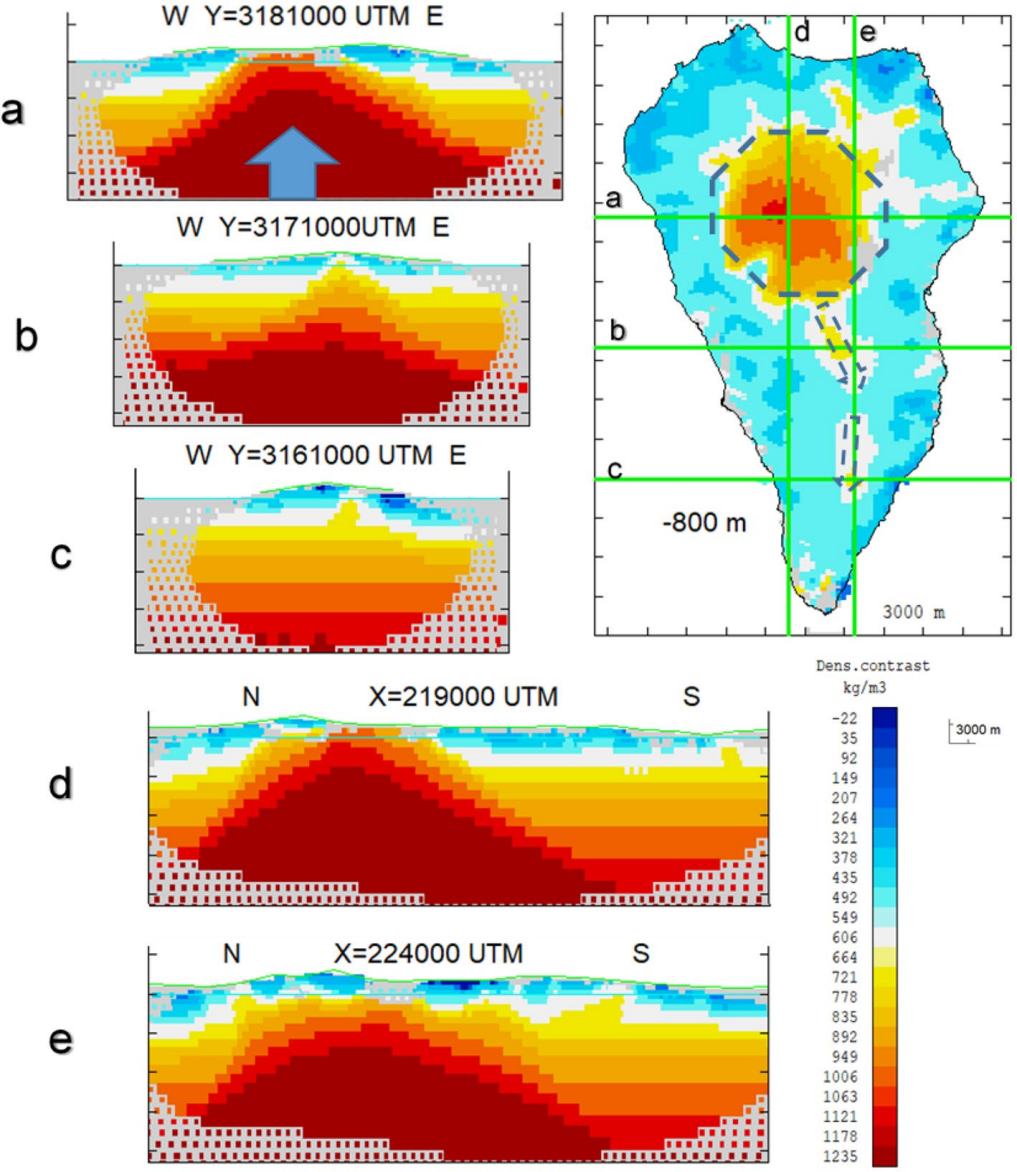
Vertical sections of the 3D model for anomalous density for La Palma Island. Panels (**a**), (**b**) and (**c**) show E-W sections. Panels (**d**) and (**e**) N-S sections. The cross lines are indicated in the horizontal section for a depth of 800 m bsl. The horizontal section includes some interpretative lines (dotted lines). Matlab software (www.mathworks.com) was used to create this figure.

**Figure 7:**
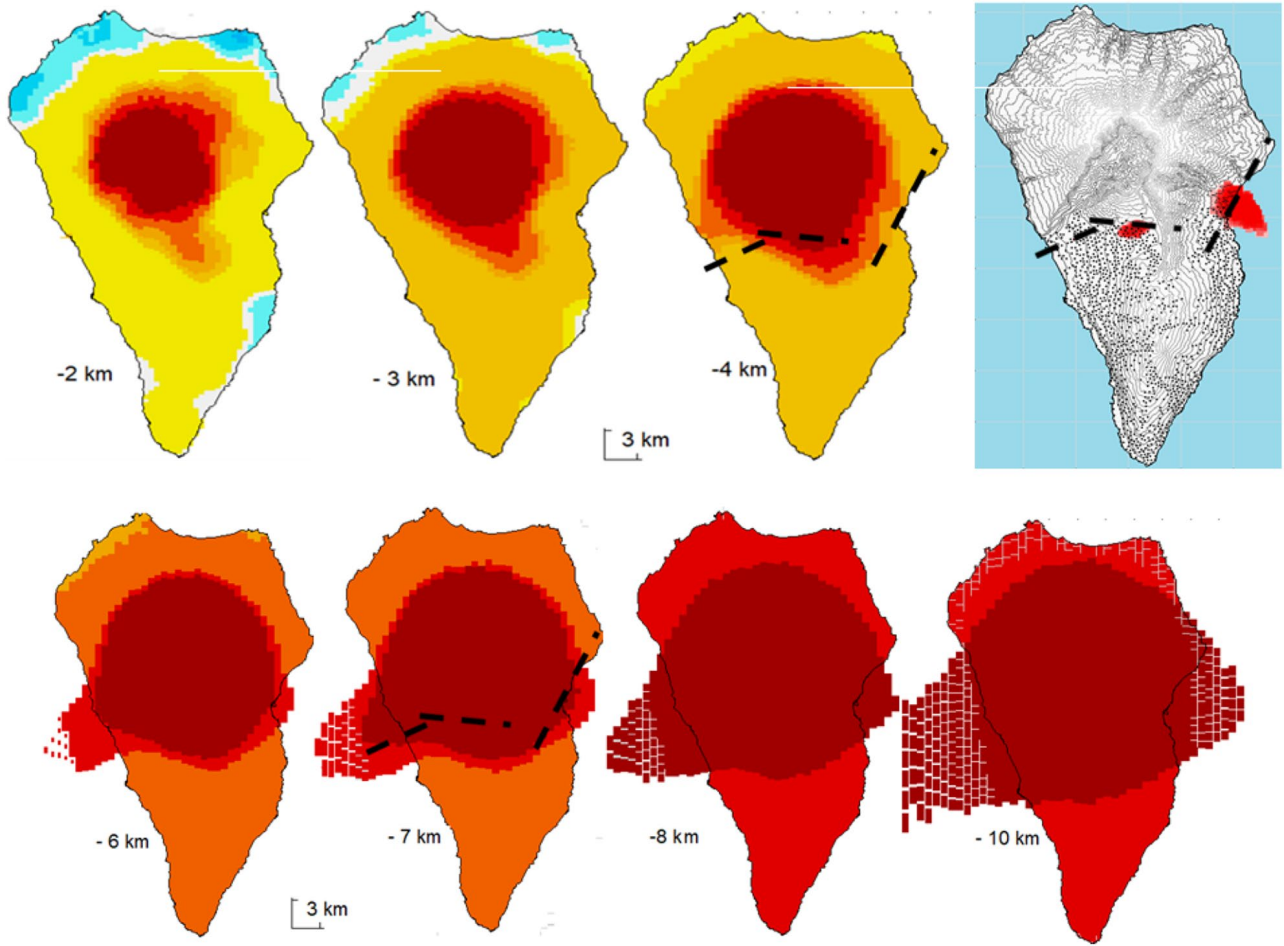
Horizontal sections of the 3D anomalous density structure and location of the 2006–2010 modeled pressure sources. Horizontal sections (from 2 to 10 km bsl) of the 3D model for anomalous density distribution for La Palma Island. Right upper panel shows the structure and the location of the modeled 2006–2010 positive pressure source E-W pattern compared with the structural one at 4 km and 7 km depth as a way of example. Matlab software (www.mathworks.com) was used to create this figure.

Using terrestrial gravity data covering the overall island^20,21^, Supplementary Fig. 6, and the methodology by Camacho et al.^25^ we obtain a 3D model of the anomalous density distribution for the crust of the island (“Methods” section). The obtained results are shown in Figs. 6, 7 and Supplementary Fig. 7.

The main feature of the inversion model is a large central body with high density located beneath the NVC (Figs. 6, 7 and Supplementary Fig. 7) and depicting the existence of a dense intrusive body highly contrasting with low density surrounding material (uncompacted erupted materials with either a higher degree of vesiculation, cracks or pores). This is the area where the radial dykes that fed the building of the submarine volcanic body and the Garafía and T-CN edifices converge^6^ and the relatively dense intrusive plutonic source body that did not reach the surface is found^8^.

The southern half of the island, consisting largely of the CV edifice (125 ka to present), is characterized by elongated minima distributed according to the main rift structures. Significant minima are detected at a depth of about 1000–2000 m bsl under the CV western slope and distributed according to en echelon portions with azimuth N130ºE. These are interpreted as associated with a slow, active process of dislocation^20^.

## Discussion

From the results, we can draw some hypotheses and conclusions about the sources responsible for the observed deformations. In the case of pressure sources, we must consider that their existence does not necessarily imply the existence of magma acting directly in the same position. The sources of relatively low magnitude positive pressure are striking due to their implications for the state of activity on La Palma.

We detected surface deformation on the Aridane Valley (Figs. 1, 2) while no deformation was detected by González et al.^26^ during 1992–2000 and 2003–2008. This aspect, coupled with other previous results^17^ which do not show anomalies before 2009, suggests that the causative source probably became active during the 2009–2010 period, indicating the onset of unrest on the island of La Palma after about 40 years of quiescence.

Complementary, a significant increase in the magmatic component of helium measured in the Dos Aguas cold spring in 2010, have been interpreted^27^ as the inputs of deep-seated CO_2_. Also soil CO_2_ efflux measurements carried out at CV volcano^27^ showed two significant increases in the CO_2_ emissions in 2011 and 2013. These variations, likely occurred with a delayed response of about one year^27^. See Supplementary Fig. 8 for the location of gases and geochemical measurements. The results obtained in 2010 and 2011 support our hypothesis of an ongoing unrest phase.

**Figure 8:**
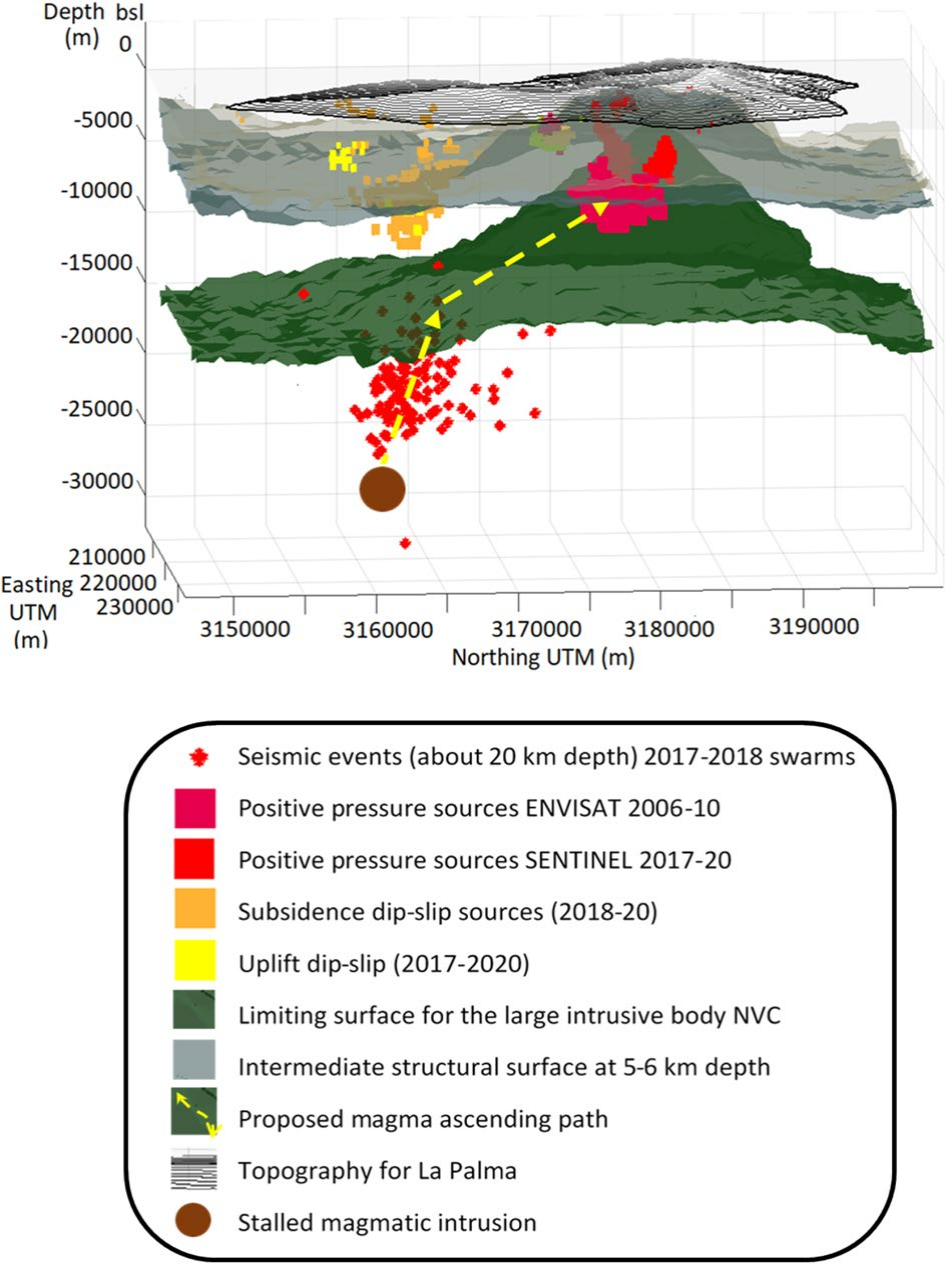
Conceptual model for the unrest evolution. Two main surfaces obtained in the 3D gravimetric crustal model (green and grey surfaces), the modeled positive pressure sources for the periods 2006–2010 and 2017–2020, the modeled dip-slip sources in 2019–2020, and the location of seismic events. The proposed path followed by the magma from depth aseismically (yellow path) before, and probably after, seismic swarms in 2017 and 2018 which may have opened new fractures. See lower inset for a description of the different components of the plot. Matlab software (www.mathworks.com) was used to create this figure.

The 2006–2010 pressure sources, Fig. 3A, are located in the central eastern sector of the island decreasing in strength (product of pressure by volume) and depth in the west direction. We observe that the western source is located in the area of the Montaña Quemada and Llano del Banco volcanoes, where recent eruptions took place (Fig. 1). This source, of low strength, is located at 2–4 km depth and has a small volume. This depth is shallower than the shallowest level of magma stagnation before historic eruptions, ~ 5 km^28–30^, where there is short-term storage weeks to days. This fact, together with the absence of an eruption since that date, and considering that a failed eruption should probably produce more detected anomalies, suggests that the shallow material was not magma.

The eastern area of this source is located at 3–10 km depth. The depth range between 6–10 km would correspond to the lower crust under La Palma, the average pre-island seafloor representing a regional horizon of neutral buoyancy for the Canary Islands magmas^31^, where magma stagnate in crustal reservoirs for years-to-decades prior to possible eruption^29^.

The depth and strength of the source in the eastern region indicate the potential intrusion of magmatic fluids from deeper areas. It is likely that the source later moved towards the west, following the NVC south limits (Figs. 3A and 7) at the same time that it ascended slightly through the crust, decreasing in strength.

Surface deformation produced by the emplacement of shallow intrusions likely occurs during unrest periods accompanied by felt seismicity^26,32^. This is not the case for the 2006–2010 time period^22,26^, when very few earthquakes occurred in the study area (Supplementary Fig. 9). However, unrest can have subtle or undetectable seismic activity as a consequence of a vent that remains open or mechanically weak following an initial stage of activity, preventing the buildup of stresses that would cause earthquakes^32^. Considering the recent volcanic activity in the area, this could be the case. Precursory seismic activity to the 1949 eruption^33^, Fig. 1, started in 1936 at Valle de Aridane, 5–10 km from the vents, and continued a few months before the eruption in the area of Fuencaliente, about 10 km to the south of Duraznero vent. The vents of Llano del Banco and Hoyo Negro opened without any preceding seismicity. Between 1936 and 1949 magma ascends from the upper mantle and stagnates and differentiates at different levels within the crust without felt seismicity^29^.

The seismic activity began after this period, with two seismic swarms in 2017 and 2018 located ~ 15 km southward of the modeled pressure sources, Supplementary Fig. 10^4,22^. Roughly speaking, earthquakes should occur close to the source feeding the unrest. However, a number of cases where the location of initial precursory earthquake swarms does not coincide with the detected sources, as for instance before the 1991 Mt. Pinatubo eruption (Philippines) and the onset of the 1995 eruption at Soufriere Hills (Montserrat), have been observed^32^. This fact is due to stress concentration in/around the volcano edifice which force earthquakes occurrence in areas which could not correspond to magma storage^32^. Therefore, we should have detected an unrest state in the same area of the 1949 San Juan eruption with similar characteristics. That unrest began at least 13 years before the eruption, with the start of precursor seismic activity^33,34^. Therefore, we could be studying a very initial phase of unrest tens of years before a possible eruption, but we must consider the possibility that it will not result in an eruption^35^.

The source has an E-W alignment pattern, Fig. 3A. Considering the 3D structure shown in Fig. 7, the source model and the structural intrusive body alignment pattern are very similar, based on the 4 km deep pressure alignment pattern. These planes, suggested by the interpretation of the DInSAR results, could be not merely topographic or delimiting volcanic units, but rather be surfaces conditioned by the large-scale morphology of the island’s intrusive dynamics and related to the feeding and unrest process detected here. It has been proposed elsewhere that these edifices are two distinct volcanoes with separate magma plumbing systems^36^ and it is possible that these surfaces mark the boundary between the extinct T/CN and the active CV volcanic edifices. Magmatic fluids could ascend from deeper zones using pre-existing fractures or contacts.

Additional modeled sources in this period are the dip-slip sources (orange color in Fig. 3A) at 2–3 km depth. We attribute these to subsidence related to the local settlement of magmatic bodies intruded at shallow depth and potentially to additional movements associated with the pressure sources. It is estimated^35^ that about 80% of the magmas that form in the oceanic intraplate environment fail to erupt. These intrusive bodies are normally denser than the volcanic rock in which they reside, so positive gravity anomalies provide information about the preferred areas of magma ascent throughout the volcanic complex’s evolution. Walker^37^ suggests that dyke swarms include the paths followed by magmas traveling through the crust, from volcanic centers along rift zones, following neutral buoyancy levels. Sheet intrusions closer to the surface (dykes and sills) are well-documented for active volcanoes, such as Kilauea or Piton de la Fournaise^38^. Cooling and crystallization of magma into swarms of intrusive dykes has been linked to cases of volcanic subsidence that have continued over years and decades^39^. They can produce some bending or initiate a rift zone^37^. Based on our gravimetric modeling results, these bodies should be hosted at a depth of about 2 km in a less dense material and we anticipate that they are responsible for the subsidence dip-slip sources in those recently active areas.

In Fig. 3A, close to the subsidence dip-slip source (orange color) and directly connected with a high pressure source (red color) there is an extended source (yellow color). We interpret it as a manifestation of the same uplift process related to the unrest in this area. This ascending dip-slip source would represent a western branch of the unrest activity.

Between both dip-slip (subsidence and uplift) sources, the adjusted model (Fig. 3A) proposes an elongated strike-slip source (green color). We interpret this as a feature of a fracture zone that separates the edge of the northern NVC complex, where we detected the unrest uplift, from the southernmost area (CV), where small subsidence phenomena take place (orange source). Another isolated strike-slip source also is observed further south (Fig. 3A) at the same depth as the dip-slip source discussed above. We interpret this as an effect, somewhat distorted, of the possible subsidence in that area. There also is a very small negative pressure source in the north, in a region with no data, and another very shallow, low strength source in the southern part of the island close to the coast. These are likely artifacts of the inversion or very local subsidence phenomena. In addition, the area covered by the dislocation sources underwent relatively recent volcanic eruptions and may be mechanically weak and easy to deform without increasing stresses that result in earthquakes.

For the period 2017–2020 (Fig. 3B), there is only one pressure source but the configuration and region are similar. The source continues extending from east to west, stopping in the central part of the island and following the same E-W alignment shown in Fig. 7. The depth range (1–8 km) is similar to the initial stages but shallower in some parts, with the maximum volume located at depths of 6–8 km and decreasing with depth.

In Fig. 3B there are again shown some dip-slip sources at 2–3 km depth. The origin is likely that discussed previously for the period 2006–2010, but their location in CV suggests that its temporal evolution should be studied, particularly as it relates to the potential landslide hazard. There also is a small, negative pressure source to the south of the island, located close to the Teneguía volcano. This source is likely associated with the measured subsidence in this area, in agreement with the previous results^26^.

Other dislocation sources that were present in the inversion results in Fig. 3A are not detected for this period. This indicates that the weak material in this area was accommodated during the previous 7 years and now the pressure variations are more important, or that the pressure effect is more significant and masks these deformations.

Looking at the time variation of the modeled sources in 2017–2020 (Figs. 4, 5), it is observed that the average positive pressure of the source increases until 2019, almost certainly connected with the two seismic swarms detected in October 2017 and February 2018, which probably opened new fractures and provided new ascent paths for magmatic gases^4^ and favoring magma rise, Fig. 5. The largest increment appears to be associated with the two seismic swarms between 2017–2018. The seismic swarms were preceded and accompanied by changes in helium and carbon dioxide emissions, suggesting magmatic intrusion beneath CV^4^. After the 2018 swarm increases in thoron concentration in soil were detected at two sites located along CV rift^4^ that can be interpreted as a magmatic intrusion from the depth and/or an active reservoir below the rift. Also, some lateral movement of magma may be associated with this seismic swarm^4^, which is consistent with the time evolution of the sources for this period. Pressure continue to increase during 2019, probably associated with aseismic recharge. As of 2019, the average pressure remains constant or decrease.

Looking at the dip-slip sources located below CV we observe that, as for the mean LOS modeled sources, during the period 2017–2018 they are not very strong. But, contrary to pressure values, they make greater and stronger as of 2019. The location of these dip-slip sources (Figs. 4 and 8) coincides with the area where most of the earthquakes of the seismic swarms are located and they started growing several months after the second swarm. These sources could be associated with fractures and, considering the existing large flank failure hazard^6^, their evolution needs to continue being monitored and interpreted.

What is the likely origin of the adjusted pressure sources? We have detected displacements with small magnitude (≤ 5 cm) changing their surface location through time (Fig. 2), as was observed in Tenerife island during the 2004–2005 volcano-tectonic crisis^1,40,41^ with the causative source evolving in space and time. As in Tenerife, we consider fluid migration at depths as a likely origin of the observed deformation, primarily in weaker areas such as the southern limits of the NVC. These are likely produced by upward migration of hydrothermal fluids from a deep magma reservoir or from the perturbation of an existing deep hydrothermal reservoir and the resultant upward movement of fluids due to magma injection. Both causes would be consistent with the gases and geochemical anomalies^4,27^ observed during the studied periods and the absence of significant broad gravity changes during 2006–2010^17^. We consider it plausible that a magma batch started to inject at approximately 8 km in depth and below and the upper part of the source shows the resultant upward movement and pressurization of fluids. Both sources would contribute to the measured deformation as well as to the gases and geochemical observed anomalies. The distribution and location of the gases and geochemical measurement anomalies^4,27^ encapsulated the migration of magma from the southern to the central part of the island, where we have the positive pressure sources.

A conceptual model for the unrest evolution that incorporates these results is presented in Fig. 8. In the onset, 2009–2010, magma started to ascend toward a shallower reservoir from the stalled magmatic intrusion located at a depth of 25–30 km^4^, along the tilted border separating the NVC and CV edifices (yellow path in Fig. 8). Magma ascended toward a depth of 8–10 km (in a weak zone where recent eruption happened), leading to an increase in the magmatic component of helium as measured in 2010 in the Dos Aguas cold spring and in CO_2_ efflux measurement at CV volcano^26^ in 2011. The magnitude of the measured displacements and the lack of significant gravity changes^17^ suggests a small volume of accumulated magma. Subsequently, in 2011–2012, when we measure deformation but do not have a high confidence in the inversion results, the input of a small quantity of magma continues, following a similar path, as reflected in the significant increases in soil CO_2_ efflux measurement at CV volcano^27^ in 2013. Again, a small quantity of magma ascending using existing fractures would be consistent with the lack of seismicity during these periods^22^ (Supplementary Fig. 9). At the beginning of the 2017–2020 period, we have two seismic swarms (October-2017, February-2018, Supplementary Fig. 10), which are associated with new paths^4^ favoring the magma ascent, reflected again in the observed geochemical signals^4^, as well as the increasing of size and importance of the dislocation sources in the CV area.

We have detected, for the first time, an initial phase of volcanic unrest in the Canary Islands using geodetic methods. We also study its evolution years after its inception, observing the changing spatial and temporal nature of activity at a potentially dangerous volcano at very high spatial resolutions and over long time periods. While these may be small and nuanced, they provide insights into the dynamic nature of the volcano.

The need to use DInSAR (with atmospheric correction and using simultaneously ascending and descending images) in La Palma as the fundamental operational tool for geodetic monitoring to detect volcanic unrest is evident, considering both, the geographic and temporal variation of the deformation. Further, its magnitude, which is at the limit of precision of the GNSS observation in campaign mode^16,17^, is clearly measurable using advanced DInSAR techniques, particularly with appropriate atmospheric correction. Much of the deformation may not be accurately detected using only observations from a GNSS network^16,17^. Nevertheless, continuous GNSS stations are a fundamental observation to provide reference points with no deformation to be used as reference locations for DInSAR^16^, as was done in this study.

Further, the results obtained from the joint inversion of ascending and descending data present a variety of sources acting simultaneously, which are not possible to detect by using classical inversion techniques and models that consider only magmatic (pressure and/or tensile) sources. This is important in La Palma, where high landslide risk can be connected with the unrest. The use of this tool allows us also to check that there are some dislocation sources acting below CV which may be associated with a future landslide and their evolution needs to continue being monitored. This demonstrates the advantage of using state-of-the-art inversion techniques that consider a large number of sources of deformation of various kinds^18,19^.

These two conclusions have a general application for the observation and interpretation of geodetic data in many volcanic areas, and, in particular for steep-sided volcanoes at oceanic islands like the Canaries.

We attribute the measured displacements and modeled sources to magma injection in small amounts at 8–10 km depth, which does not produce important displacements, but results in an upward movement and/or pressurization of fluids at shallower depths and activated dislocation sources around the pressure ones and below CV volcano. This unrest is similar in many aspects to the one preceding the 1949 eruption and, for some features, to that observed at Tenerife in 2004–2005. It is completely different from that seen at El Hierro and Teneguia eruptions. The observed differences and similarities are important to discriminate between the possible types of unrest in the Canaries, and to distinguish the likelihood that an unrest process could culminate with a future eruption (or not), the possible impact on the stability of CV, and its likely duration compared with previous stages of unrest in La Palma and in the Canary Islands. Therefore, it is of critical importance to continue monitoring the current evolution of the unrest, particularly considering the recent increase in seismic activity on the island^22^, Supplementary Fig. 10.

## Methods

### Differential interferometric synthetic aperture radar (DInSAR)

In this work three different time periods are studied: 2006–2010 using images from ENVISAT satellite, 2009–2016 using RADARSAT-2 images and 2017–2020 using images from Sentinel-1 constellation, formed by Sentinel-1A and 1B satellites. For ENVISAT and Sentinel-1 satellites, ascending and descending orbits are considered while RADARSAT-2 only descending orbit had enough data to perform a DInSAR study. The Single Look Complex (SLC) images were provided by ESA (ENVISAT and Sentinel-1) and have been downloaded from the Alaska Satellite Facility (ASF) while RADARSAT-2 acquisitions were provided by the Canadian Space Agency (CSA). For ENVISAT and RADARSAT-2, all the available acquisitions were processed, however, some of them were discarded due to their high temporal or spatial baselines or high doppler differences. Sentinel-1 acquisitions for La Palma island in Interferometric Wide (IW) mode started in July 2017 and we choose a 12-day frequency for this study. The complete lists of SLC images used in this work are available in Supplementary Tables 1–3.

ENVISAT and Sentinel-1 datasets were processed using SUBSIDENCE-GUI interferometric software^21^ with similar parameters. Before the interferometric generation, all images for each orbit were registered to a common reference image. The reference image was selected to minimize perpendicular and temporal baselines across the dataset to minimize registration errors. Check Supplementary Tables 1–3 for the reference images.

Interferogram pairs were generated by double minimum criteria, avoiding those with high temporal and perpendicular baselines. With the double minimum selection, we select one subset that is sensitive to deformation (large temporal baselines) and less sensitive to Digital Elevation Model (DEM) errors (small spatial baselines). The other subset behaves just the contrary, sensitive to DEM error (large spatial baselines) and less sensitive to deformation (short temporal baselines). A total of 204 interferograms were generated for ENVISAT and 1063 for Sentinel-1. The higher number of Sentinel-1 interferograms is due to its small temporal and spatial baselines. To remove the topographic phase from the interferograms, an external high-resolution DEM has been used. The respective DEMs were generated using MDT025 and MDT05 data from the Instituto Geográfico Nacional (IGN)^22^, with 25 m resolution for ENVISAT, and 5 m resolution for RADARSAT-2 and Sentinel-1 respectively.

For the period 2010–2017, we used RADARSAT-2 images to estimate annual mean velocities. This dataset consists of 25 Wide-Fine and 13 Fine beam descending orbit SLC images and 63 interferograms. The interferograms were computed with GAMMA software^42^. Four lines by eight samples multilooking was applied and geocoded products were produced with a ground resolution of approximately 40 m. Multilooked differential interferograms were filtered using adaptive filtering with filtering function based on the local fringe spectrum^43^ and unwrapped using the minimum cost flow algorithm^44^. Baseline refinement was performed to remove residual orbital ramps followed by a minor interpolation of each interferogram to increase the coverage reduced by decorrelation. The linear rate and time series of LOS were computed with MSBAS software^24^ using pixels with coherence above 0.5 in all interferograms.

To retrieve surface displacement from differential interferograms, an important noise phase component caused by the Atmospheric Phase Screen (APS) has been mitigated. Generally, the magnitude of APS cannot be ignored because they can often be comparable, or even larger to the interested geophysical signals^14,15^. Due to the strong variation of topography and an extensive coastline, La Palma is prone to suffer APS. In this paper, three APS mitigation methods have been used. The first one is the classic temporal and spatial filtering, and the second one is based on external weather forecast data. In the case of ENVISAT, in order to separate APS and the non-linear velocity, two filters were applied: a spatial low pass filtering with a 1-km correlation window and a high pass temporal filtering with a window of 60 days and a minimum of 4 temporal samples. After this processing, the non-linear displacement can be calculated. For the period 2010–2017 and RADARSAT-2 data the APS correction was computed based on a regression analysis of the observed phase and elevation and subtracted from the interferograms. This method is not very reliable in this region because of the predominant wind direction that keeps moisture on one side of the island. For the Sentinel-1 dataset, to correct APS as much as possible, the realistic integration D-LOS algorithm has been used based on the latest ERA5 global reanalysis data. It has been demonstrated^14,15^ that the D-LOS shows a great improvement compared to the conventional zenith-based method. Besides, among different weather forecast datasets, it is also demonstrated that the ERA5 data from ECMWF performs the best^15^. After applying the D-LOS algorithm, APSs have been mitigated significantly from the original interferograms. Supplementary Fig. 1 shows a typical example (Sentinel-1 interferogram 20,180,914–20,180,926 pair) of APS correction. The original interferogram (Supplementary Fig. 1A) shows a significant APS phase component related to the topography. The estimated APS shown in Supplementary Fig. 1B presents a very similar pattern. Consequently, the residue phase (Supplementary Fig. 1C) after APS correction is very smooth with a small variation.

To calculate the surface displacements SUBSIDENCE-GUI was used again, the software implementation of the Coherent Pixel Technique (CPT) algorithm. This method uses distributed scatters at low-resolution over the multi-looked interferograms, similar to the wide-used Small Baselines Subset (SBAS). Because of the characteristics of La Palma (a volcanic island with strong topography and high density of vegetation), this kind of analysis produces better results than a full-resolution method like the permanent scatters (PS) approach^45^. To evaluate the phase quality of the pixels a mean coherence map has been processed using a multilook window of twenty-five samples in range and five lines in azimuth for Sentinel-1, while for ENVISAT the window is twenty-five lines by five samples. This process results in lower resolution pixels calculated from an average of 125 pixels from the original interferogram. The multilooked interferograms have pixels with a ground resolution of about 100 m × 100 m.

A coherence criterion has been chosen to select those pixels with enough phase quality to obtain surface deformation. Different thresholds of a medium coherence have been used for ENVISAT and Sentinel-1: 0.35 for ENVISAT and 0.4 for Sentinel-1, corresponding to a phase standard deviation of 12º and 9º respectively. These values provide good spatial coverage and enough phase quality to obtain a convergent solution. To fix the floating solutions of the linear velocity and DEM error, CPT uses points of known velocity and altitude for the studied period, also called seeds. Two seeds have been selected, one on the east coast of the island and another on the west coast considering their stability determined by GNSS observations^46^. See also the next subsection. See Supplementary Fig. 1A for seeds locations.

### Global navigation satellite system (GNSS)

Ground deformations of La Palma are currently provided by few sparse continuous GNSS stations (Supplementary Fig. 2). We collected all the available free raw data from IGN^47^ and Grafcan^48^ websites. To improve the overall configuration of the network and tie the regional measurements to an external global reference frame, data coming from all the continuous GNSS stations installed on the Canary archipelago as well as other 15 continuously operating global tracking stations, largely from the IGS^49^ and EUREF^50^ permanent networks, were introduced in the processing. The GNNS phase observations were processed by using the GAMIT/GLOBK 10.71 software^51^ and by adopting the IGS “Repro2 campaign” standards^52^. To eliminate phase biases related to drifts in the satellite and receiver clock oscillators, an ionosphere-free linear combination of GNSS phase observables was adopted during the processing, by applying a double differencing technique. The GNSS phase data were weighted according to an elevation-angle-dependent error model^51^ using an iterative analysis procedure whereby the elevation dependence was determined by the observed scatter of phase residuals. In this analysis, the parameters of the satellites’ orbit were fixed to the IGS final products (https://cddis.nasa.gov/archive/gnss/products/). IGS absolute antenna phase center models for both satellite and ground-based antennas were adopted in order to improve the accuracy of vertical site position component estimations^53^. The first-order ionospheric delay was eliminated by using the ionosphere-free linear combination, while second-order ionospheric corrections^54^ were applied using the IONEX files from the Center for Orbit Determination in Europe (CODE). The tropospheric delay was modeled as a piecewise linear model and estimated using the VMF1^55^ with a 10° cutoff. The Earth Orientation Parameters (EOP) were tightly constrained to prior values obtained from IERS Bulletin B. The ocean tidal loading was corrected using the FES2004 model^56^. The International Earth Rotation Service (IERS) 2003 model for diurnal and semidiurnal solid Earth tides was also adopted. The results of this processing step are daily estimates of loosely constrained station coordinates, and other parameters, along with the associated variance–covariance matrices.

In a successive step, the loosely constrained daily solutions were used as quasi observations in a Kalman filter (GLOBK) in order to estimate a consistent set of daily coordinates (i.e. time series) for all sites involved. Each time series was analyzed for linear velocities, periodic signals and antenna jumps by using the TSVIEW software package described by Herring et al.^57^. In order to obtain clean time-series, any position estimate whose uncertainty was greater than 20 mm or whose value differed by more than 10 mm from the best-fitting linear trend was removed. By using the GLORG module of GLOBK, the daily solutions and their full covariance matrices were combined to estimate a consistent set of positions and velocities in the ITRF14 reference frame^58^. By considering a set of GNSS stations distributed over the whole archipelago, we estimated the Euler vector components (latitude and longitude of a pole, rotation rate) for a local reference frame in order to highlight eventual local ground deformation pattern. Resulting Euler vector components are: latitude, 49.576º ± 0.306º; longitude, -85.652º ± 4.526º; rotation rate, 0.263144º ± 0.008943º.

### Direct modeling and inversion technique

We modeled the LOS DInSAR (both ascending and descending, or just descending) deformation field by using a recently developed methodology^19^ which has been applied to model the simulated and real study cases. This original methodology carries out a multi-source inversion of ground deformation data to better understand the subsurface causative processes. Roughly speaking, the observation data, contain the direct effect at the surface of the deformation caused by an arbitrary combination of 3D pressure and generic dislocation sources located at different depths in the medium. The proposed method identifies a combination of pressure bodies and different types of dislocation sources (dip-slip, strike-slip and tensile) that can represent magmatic sources and other processes such as earthquakes, landslides or groundwater-induced subsidence through the aggregation of elemental cells. A nonlinear approach permits the determination of the location, size and three-dimensional configuration, without any a priori assumption, of the potential causative sources. The 3D geometry of the sources is obtained as an aggregation of elementary sources.

This approach has the following main features: (1) simultaneous inversion of the deformation components and/or LOS data; (2) simultaneous determination of different structures such as pressure bodies or dislocation sources, representing local and regional effects; (3) a fully 3D context; and (4) no initial hypothesis about the number, geometry or types of the causative sources is necessary.

For the pressure sources, the method employs the formulation presented by Geertsma and Van Opstal^59^, which is more rigorous than one based on the well-known Mogi^60^ formalism. For the calculation of the effects of surface deformation produced by elementary sources (tensile, strike and dip), the formulation of Okada^61^ is implemented. See Camacho et al.^19^ for a more detailed description of the inversion methodology.

### Structural gravimetry

As previously described, a terrestrial gravity survey composed of 317 benchmarks covering the island of La Palma was carried out in 2006^20,27^ (Supplementary Fig. 6). The corresponding elevation values were simultaneously determined by GNSS observations, supplemented by a 25-m resolution digital elevation model (DEM) for the island. For the surrounding marine areas, satellite altimetry data was used to provide information about the gravity anomaly, geoid and bathymetry.

Camacho et al.^20^ used these gravimetric data to develop a model of anomalous density structures on La Palma using a semi-automatic inversion process. This model assumes that the anomalous bodies are in a homogeneous environment, and that, in addition to adjusting the observed data, they verify the usual conditions of regularity. In a later work^25^ they extended the inversion methodology to the case of considering stratified structures, with an increase in density with depth. More realistic anomalous models are obtained using this approach.

Here we applied the latest, improved methodology to the gravimetric data of the island of La Palma to update the structural framework results. We first divided the subsurface volume into a partition of small prismatic cells. We considered approximately 90,000 cells with an average side of 450 m that cover the subsurface volume to a depth of 12,000 m with sufficient resolution. In addition, we considered a regular layering in sub-horizontal layers (12 layers, arbitrarily) of increasing density in depth. Finally, we chose an adequate value of the regularity parameter to obtain a null auto-correlation of the final residuals^20^. Once these parameters are set, the inversion process is automatic and a 3D model of anomalous densities is obtained.

## Supplementary Information

Supplementary Information

## Data Availability

SLC data from ENVISAT and Sentinel-1 is publicly available on the Alaska Satellite Facility website: www.asf.alaska.edu. RADARSAT-2 data is available upon request to the Canadian Space Agency. LOS displacement data sets are available upon request to the authors.
