# Supplementary material for: Detection of volcanic unrest onset in La Palma, Canary Islands, evolution and implications
Source: Sci Rep. 2021 Jan 28;11:2540. doi: 10.1038/s41598-021-82292-3 (PMC7844277; doi:10.1038/s41598-021-82292-3)
Supplement: Supplementary file 1 — Supplementary Information [file 41598_2021_82292_MOESM1_ESM.pdf]

Supplementary Information for

Detection of volcanic unrest onset in La Palma, Canary Islands, evolution and implications

José Fernández[1*], Joaquín Escayo[1], Zhongbo Hu[2], Antonio G. Camacho[1], Sergey V. Samsonov[3], Juan F. Prieto[4], Kristy F. Tiampo[5], Mimmo Palano[6], Jordi J. Mallorquí[2], Eumenio Ancochea[7]

[1]Instituto de Geociencias (CSIC, UCM). Calle del Doctor Severo Ochoa, nº 7. Ciudad Universitaria. 28040-Madrid, Spain.

[2]CommSensLab-Universitat Politècnica de Catalunya, D3-Campus Nord-UPC, C. Jordi Girona 1-3, 08034, Barcelona, Spain.

[3]Canada Centre for Mapping and Earth Observation, Natural Resources Canada, 560 Rochester Street, ON K1A 0E4, Ottawa, Canada.

[4]ETS de Ingenieros en Topografía, Geodesia y Cartografía, Universidad Politécnica de Madrid, 28031-Madrid, Spain.

[5]Cooperative Institute for Research in Environmental Sciences (CIRES), 216UCB, University of Colorado at Boulder, Boulder, CO, 80309, USA.

[6]Istituto Nazionale di Geofisica e Vulcanologia, Osservatorio Etneo - Sezione di Catania, Piazza Roma 2, 95125 Catania, Italy.

[7]Departamento de Mineralogía y Petrología, Fac. CC. Geológicas, Universidad Complutense de Madrid, 28040 Madrid, Spain.

*Correspondence to: jft@mat.ucm.es

This PDF file includes:

Supplementary Figs. 1 to 10
Supplementary Tables 1 to 3

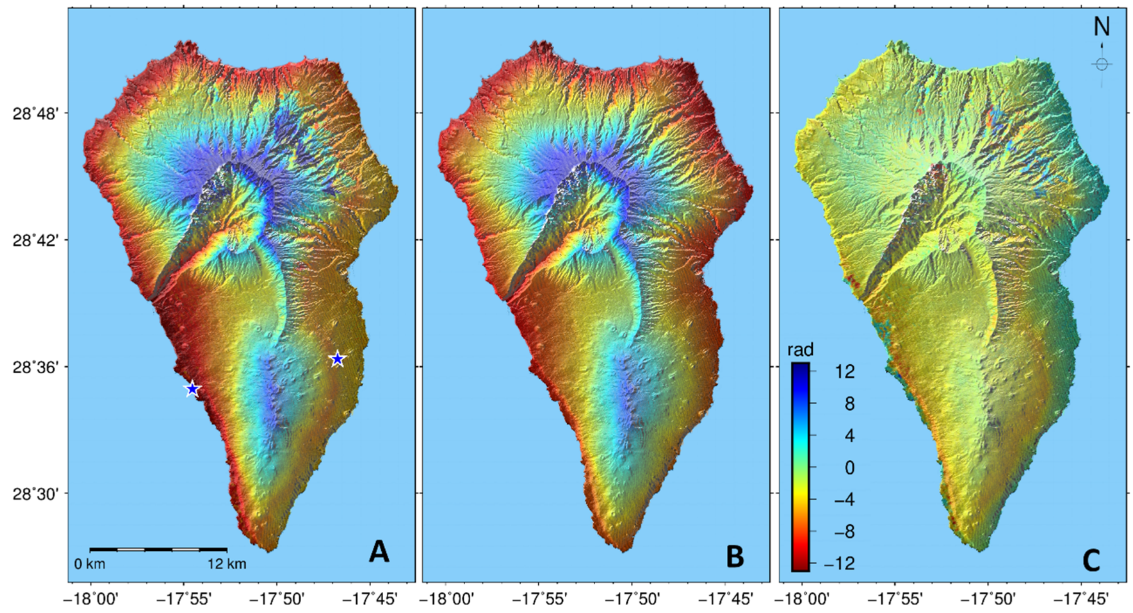

Supplementary Figure 1: Example of APS correction for a Sentinel-1 interferogram (interferogram 20180914-20180926). **(A)** shows the original unwrapped interferogram where star symbols represent the points used as velocity seeds, (**B**) is the estimated atmospheric screen phase, and (**C**) is the residual phase after subtracting the APS from the original interferogram. GMT software (www.generic-mapping-tools.org) software was used to create this figure.

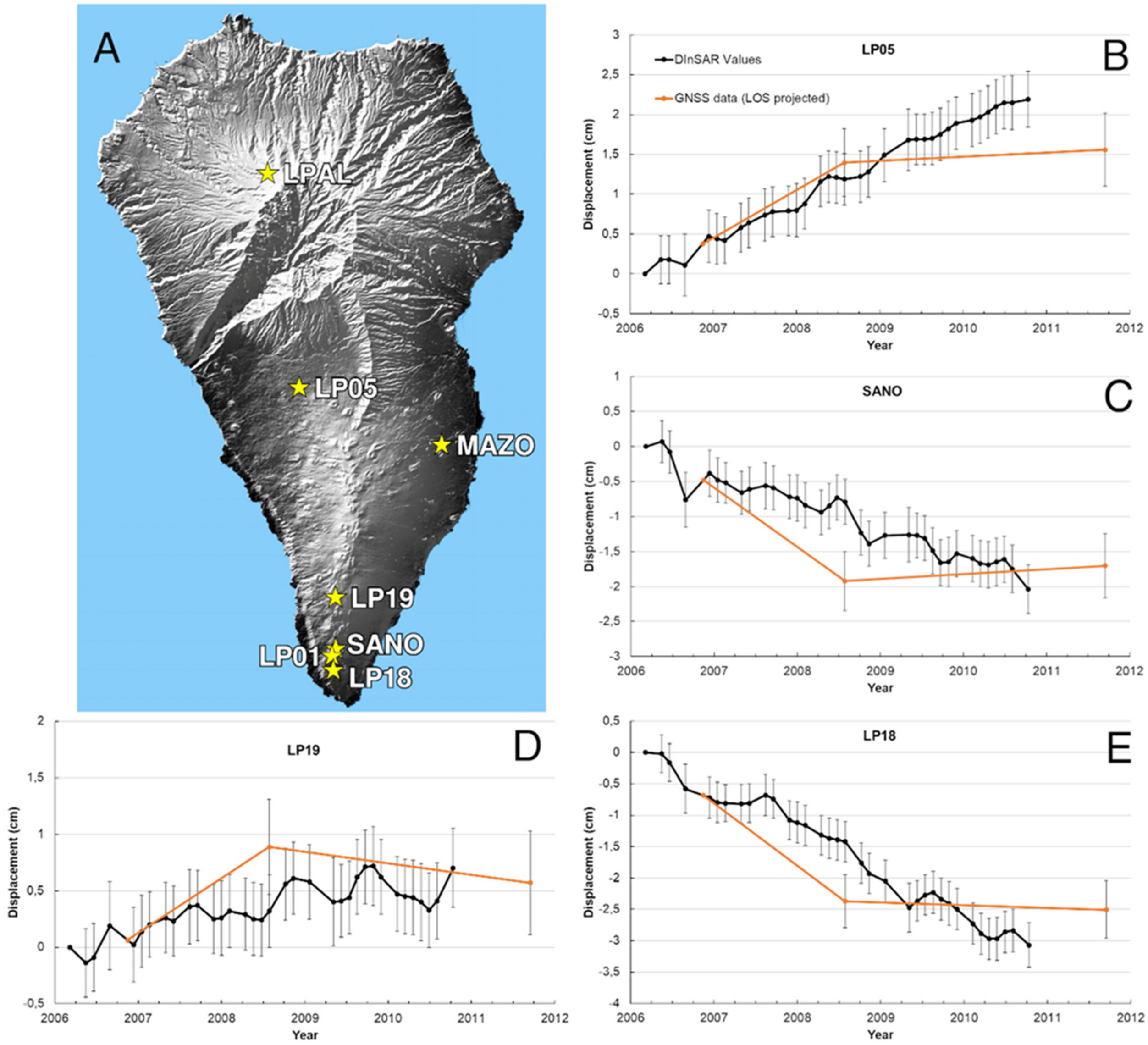

Supplementary Figure 2: Comparison between DInSAR time-series and the GNSS LOS projected values for several stations and survey points. (**A**) Location of the stations used in the comparison, (**B-E**) GNSS LOS projected data and ENVISAT descending time series of four different stations: LP05, SANO, LP18 and LP19[1]. (**F**) Time series obtained for Sentinel-1 ascending data plotted with GNSS LP01 station LOS projected values. (**G**) Time series for Sentinel-1 descending data plotted with GNSS LP01 station LOS projected values. (**H**) Time series for Sentinel-1 descending data plotted with GNSS LPAL LOS projected values. GMT software (www.generic-mapping-tools.org) and Microsoft Excel 365 were used to create this figure. (continues in the next page)

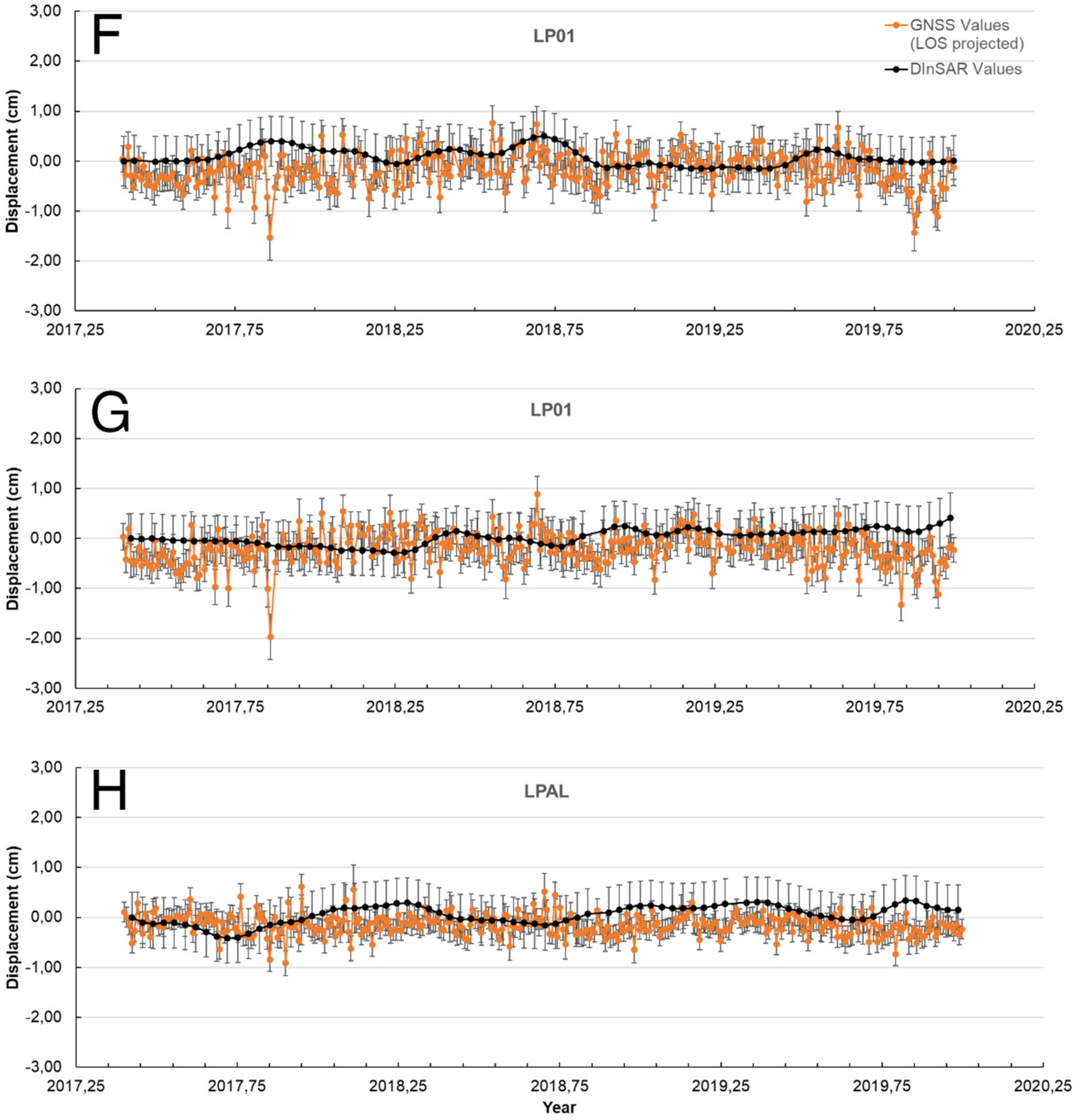

Supplementary Figure 2 (continuation).

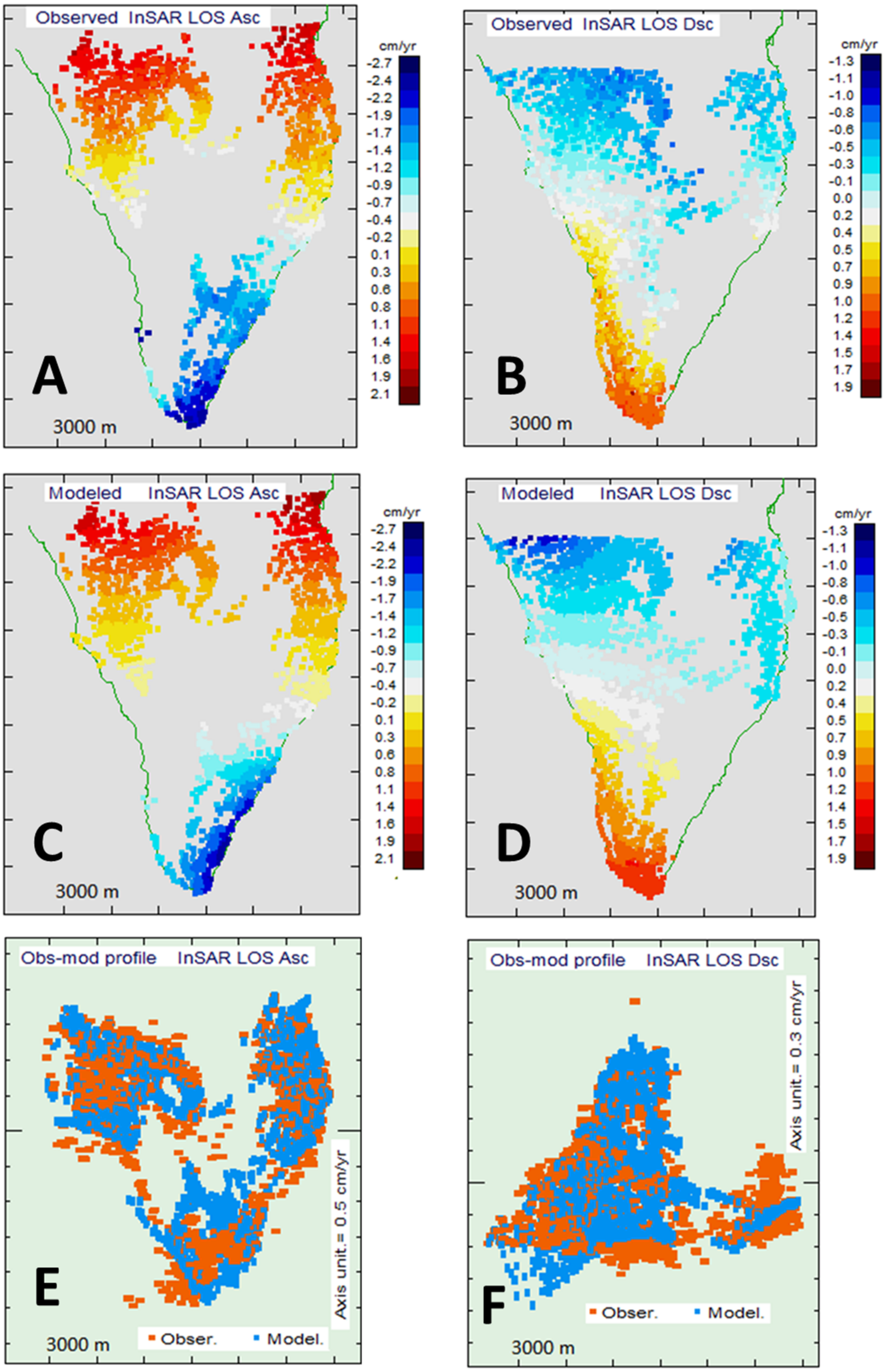

Supplementary Figure 3: 2006-2010 observed mean LOS velocity displacement values determined using (**A**) ascending and (**B**) descending ENVISAT radar images. Modeled ascending (**C**) and descending (**D**) LOS. Observed-modeled comparison of ascending (**E**) and descending (**F**) LOS velocity displacements. Matlab software (www.mathworks.com) was used to create this figure.

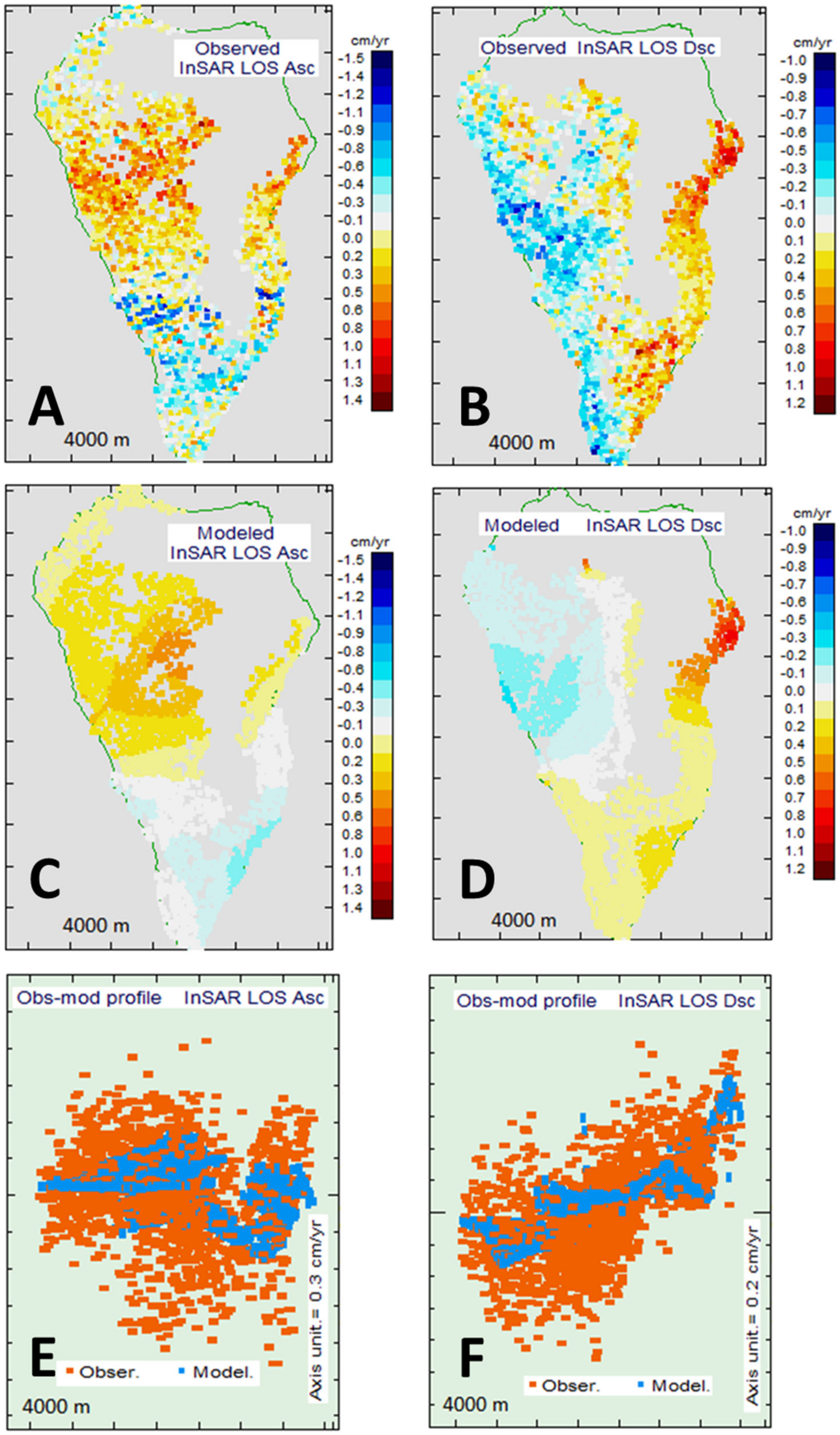

Supplementary Figure 4: 2017-2020 observed mean LOS velocity displacement values determined using (**A**) ascending and (**B**) descending Sentinel-1 radar images. Modeled ascending (**C**) and descending (**D**) LOS. Observed-modeled comparison of ascending (**E**) and descending (**F**) LOS velocity displacements. Matlab software (www.mathworks.com) was used to create this figure.

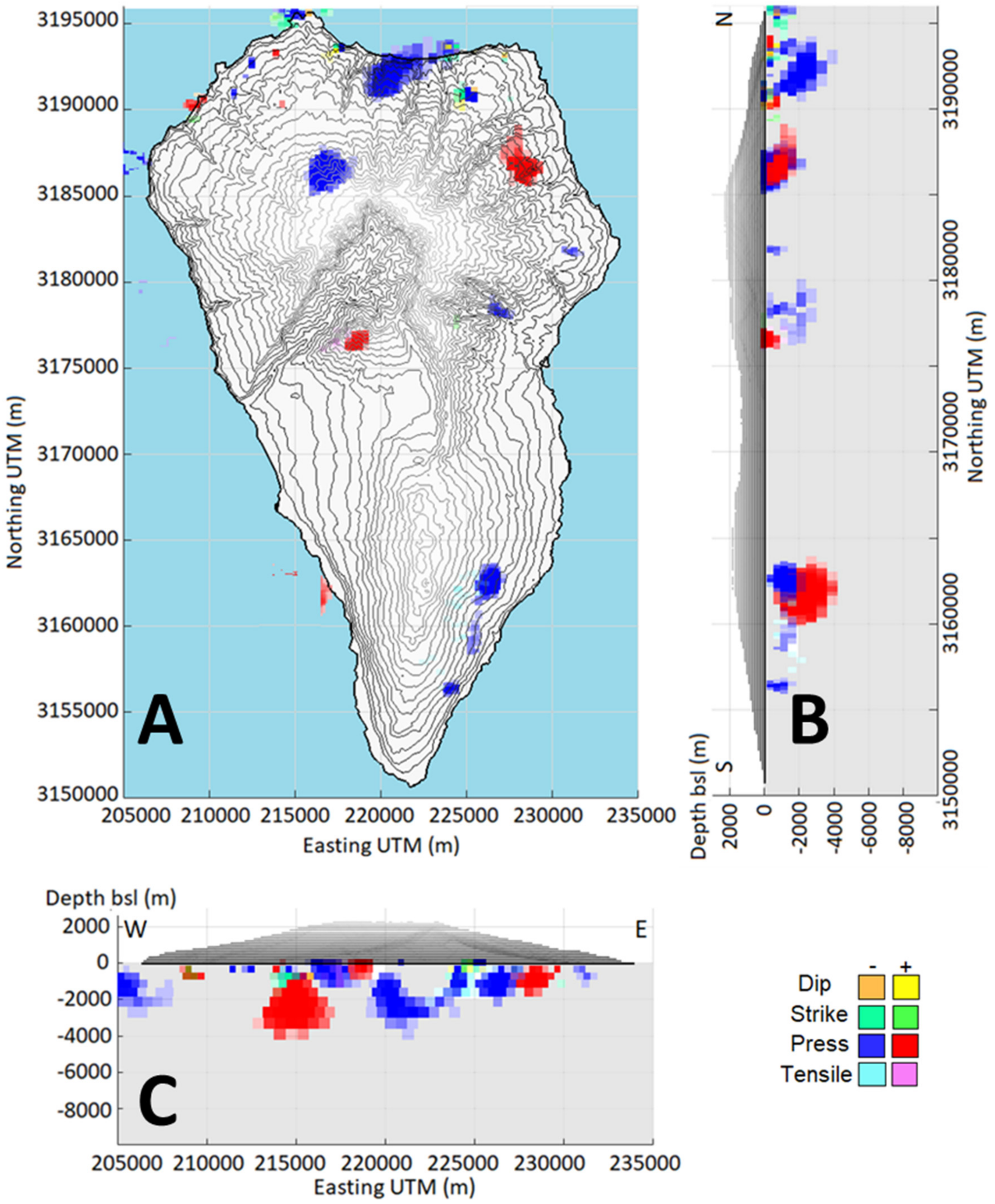

Supplementary Figure 5: (**A**) Planar, (**B**) NS and (**C**) WE vertical elevation views of the significant sources adjusted for the period 2010-2017. See color code for type of source and intensity. Dots denote location of ascending and descending LOS deformation data. Matlab software (www.mathworks.com) was used to create this figure.

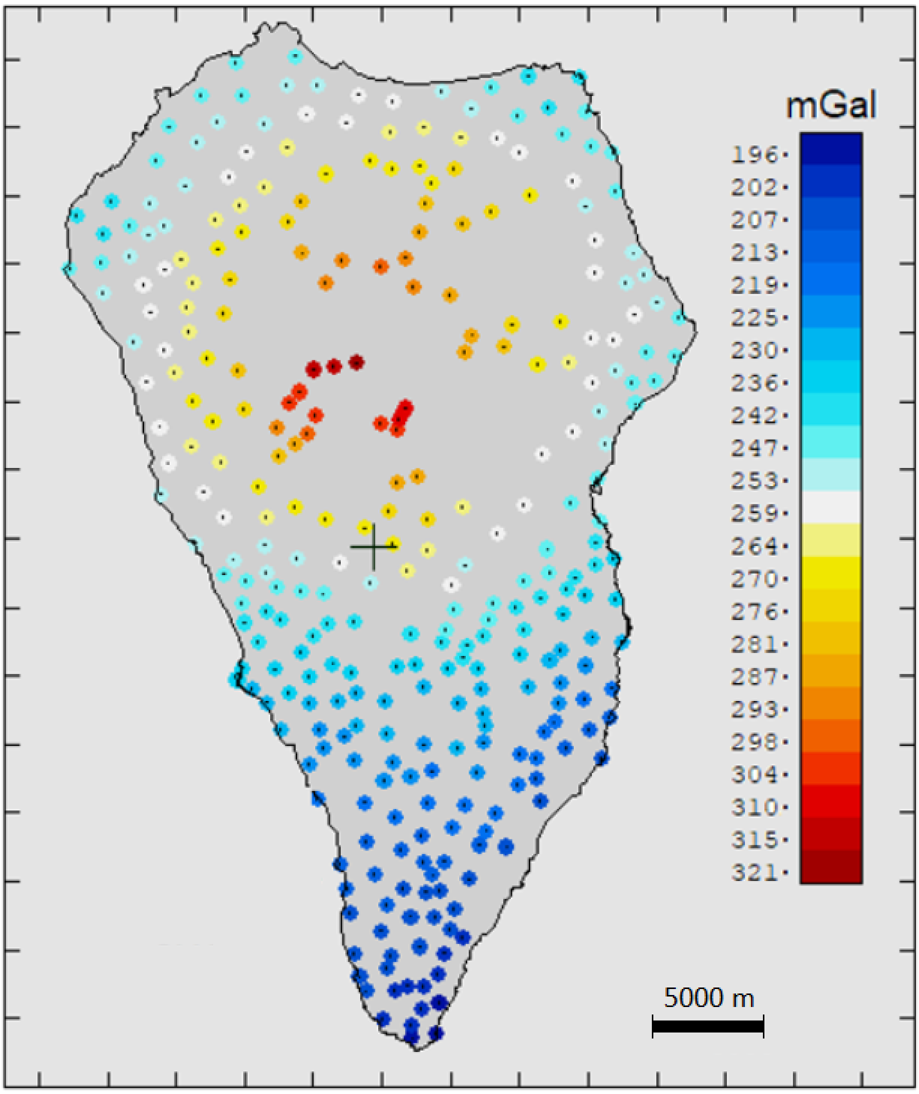

Supplementary Figure 6. Location of gravimetric stations on La Palma island and gravimetric anomaly map. The location of the 317 gravimetric stations is marked by the color dots. Color scale gives the values of the gravimetric anomaly (mGal) corrected from topography effect. Gravimetric data[30] was collected in 2006 specifically for the structural study. Matlab software (www.mathworks.com) was used to create this figure.

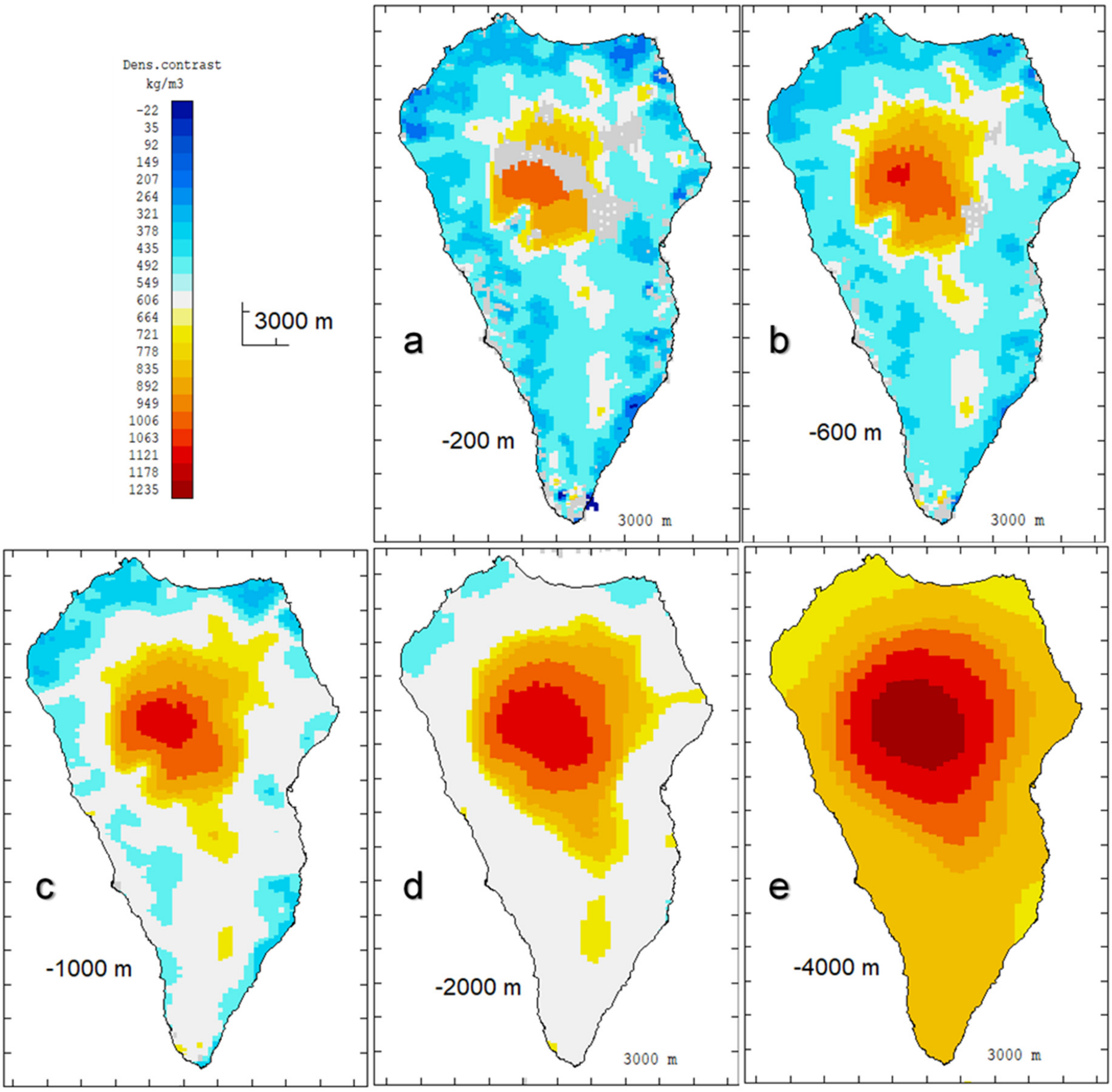

Supplementary Figure 7: Horizontal sections of the 3D model for anomalous density for La Palma at selected depths: (**a**) 200 m asl, (**b**) 600 m bsl, (**c**) 1,000 m bsl, (**d**) 2000 m bsl, and (**e**) 4,000 m bsl. Matlab software (www.mathworks.com) was used to create this figure.

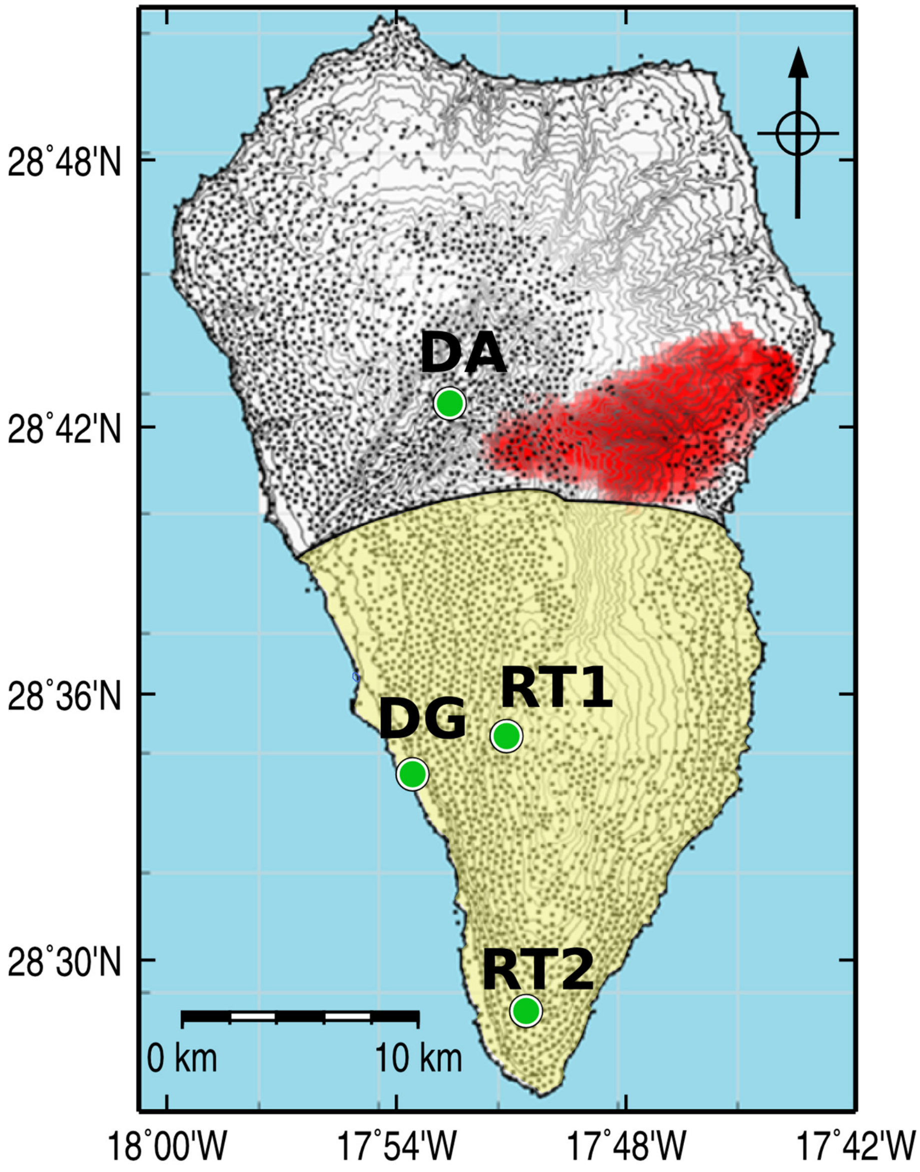

Supplementary Figure 8: Approximate location of the area covered by the soil CO_2 efflux survey carried out at Cumbre Vieja volcano marked with green lines[2], and location of the geochemistry observation stations[3] (Radon/Thoron stations (RT1 and RT2), the dissolved gas station (DG) and the Dos Aguas (DA) cold spring where the free gas station is located), superimposed on the image of the island with the average source of the period 2017-2020. Matlab software (www.mathworks.com) and GMT software (www.generic-mapping-tools.org) were used to create this figure.

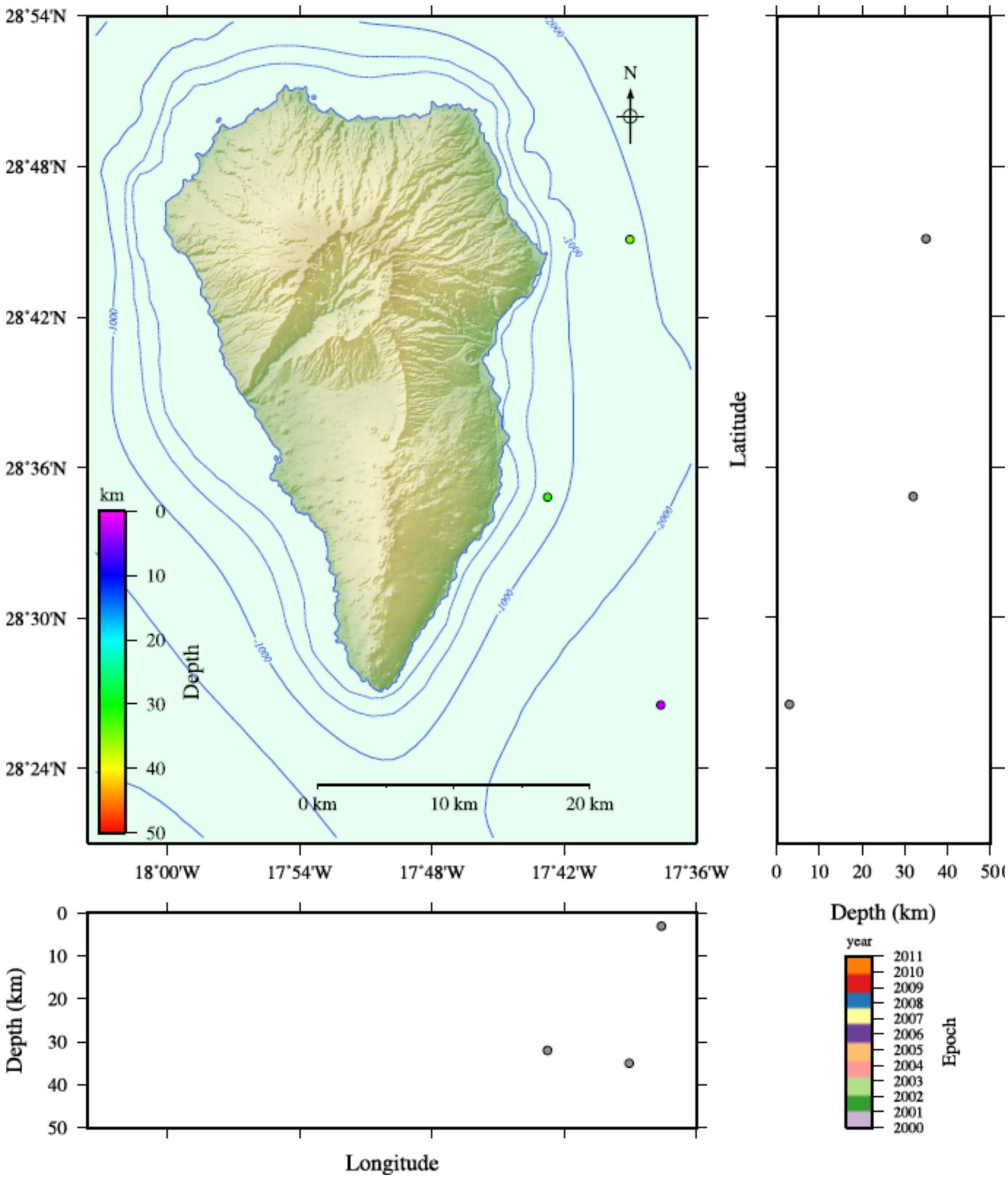

Supplementary Figure 9: Seismicity under La Palma Island during the period January 2000-December 2010. GMT software (www.generic-mapping-tools.org) was used to create this figure.

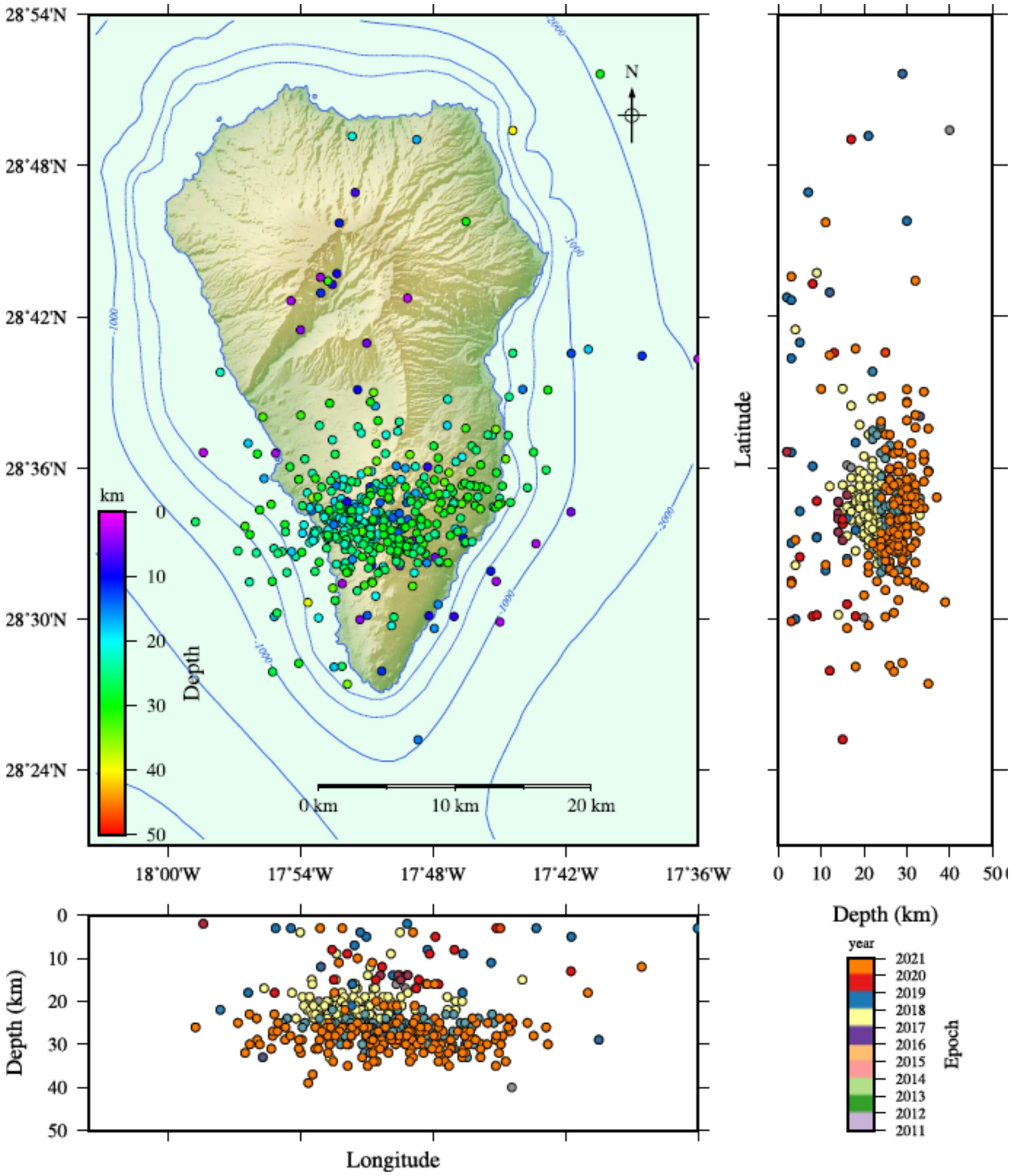

Supplementary Figure 10: Seismicity under La Palma Island during the period January 2011-August 2020. GMT software (www.generic-mapping-tools.org) was used to create this figure.

ENVISAT									
ASCENDING				DESCENDING					
#	DATE	#	DATE	#	DATE	#	DATE	#	DATE
1	~~*20040721*~~	14	**20070711**	1	~~*20031013*~~	15	20070604	29	20090504
2	20060308	15	20070815	2	~~*20040301*~~	16	20070813	30	20090608
3	20060412	16	20070919	3	~~*20040719*~~	17	20070917	31	20090713
4	20060621	17	20071128	4	20060306	18	20071126	32	20090817
5	~~*20060726*~~	18	~~*20080102*~~	5	20060515	19	20071231	33	20090921
6	20060830	19	~~*20080206*~~	6	20060619	20	**20080204**	34	20091026
7	20061004	20	20080416	7	~~*20060724*~~	21	20080414	35	20091130
8	20061108	21	20080521	8	22060828	22	20080519	36	20100208
9	~~*20070117*~~	22	20080730	9	~~*20061002*~~	23	20080623	37	20100315
10	20070221	23	~~*20080903*~~	10	~~*20061106*~~	24	20080728	38	20100419
11	20070328	24	20081008	11	20061211	25	20081006	39	20100524
12	20070502	25	20090121	12	20070115	26	20081110	40	20100628
13	20070606	26	20090401	13	20070219	27	20090119	41	20100802
				14	20070430	28	~~*20090330*~~	42	20101011

Supplementary Table 1: ENVISAT SLC acquisition dates used in this study. In italics and strikethrough, the images that were discarded due to their long baselines or large Doppler differences. The SLC images used as reference for coregistration are in bold.

RADARSAT-2					
DESCENDING					
Wide-Fine Beam				Fine Beam	
#	DATE	#	DATE	#	Date
1	20110803	15	20141022	1	20091107
2	20120517	16	20150408	2	20100331
3	20121101	17	20150619	3	20100705
4	20131027	18	20150713	4	20100915
5	20131214	19	20160214	5	20101220
6	20140107	20	20160309	6	20110326
7	20140413	21	20160402	7	20110606
8	20140507	22	20160824	8	20110630
9	20140531	23	20160917	9	20110910
10	20140624	24	20161104	10	20111215
11	20140718	25	20161128	11	20121209
12	20140811	26	20161222	12	20130315
13	20140904			13	20130923
14	20140928			14	20131017

Supplementary Table 2: Acquisition dates of the RADARSAT-2 SLCs used in this study. Processing was performed pairwise without primary reference image to improve coregistration precision.

Sentinel-1											
Ascending						Descending					
**#**	**Date**	**#**	**Date**	**#**	**Date**	**#**	**Date**	**#**	**Date**	**#**	**Date**
1	20170526	31	20180602	61	20190603	1	20170603	31	20180529	61	20190617
2	20170607	32	20180614	62	20190621	2	20170615	32	20180610	62	20190629
3	20170701	33	20180626	63	20190703	3	20170627	33	20180622	63	20190711
4	20170713	34	20180708	64	20190715	4	20170709	34	20180704	64	20190723
5	20170725	35	20180720	65	20190727	5	20170721	35	20180716	65	20190804
6	20170806	36	20180801	66	20190808	6	20170802	36	20180728	66	20190816
7	20170818	37	20180813	67	20190820	7	20170814	37	20180809	67	20190828
8	20170830	38	20180825	68	20190901	8	20170826	38	20180821	68	20190909
9	20170911	39	20180906	69	20190913	9	20170907	39	20180902	69	20190921
10	20170923	40	20180918	70	20190925	10	20170919	40	20180914	70	20191003
11	20171005	41	20180930	71	20191007	11	20171001	41	20180926	71	20191015
12	20171017	42	20181012	72	20191019	12	20171013	42	20181008	72	20191027
13	20171029	43	20181024	73	20191031	13	20171025	43	20181020	73	20191108
14	20171110	44	20181105	74	20191112	14	20171106	44	20181101	74	20191120
15	20171122	**45**	**20181117**	75	20191124	15	20171118	**45**	**20181125**	75	20191202
16	20171204	46	20181129	76	20191206	16	20171130	46	20181207	76	20191214
17	20171216	47	20181211	77	20191218	17	20171212	47	20181219	77	20191226
18	20171228	48	20181223	78	20191230	18	20171224	48	20181231	78	20200107
19	20180109	49	20190104	79	20200111	19	20180105	49	20190112	79	20200119
20	20180121	50	20190116	80	20200123	20	20180117	50	20190124	80	20200131
21	20180202	51	20190128	81	20200204	21	20180129	51	20190205	81	20200212
22	20180214	52	20190209	82	20200216	22	20180210	52	20190217	82	20200224
23	20180226	53	20190221	83	20200228	23	20180222	53	20190301	83	20200307
24	20180310	54	20190305	84	20200311	24	20180306	54	20190313	84	20200319
25	20180322	55	20190317	85	20200323	25	20180318	55	20190325	85	20200331
26	20180403	56	20190329	86	20200404	26	20180330	56	20190406	86	20200412
27	20180415	57	20190410	87	20200416	27	20180411	57	20190430	87	20200424
28	20180427	58	20190428	88	20200428	28	20180423	58	20190512	88	20200506
29	20180509	59	20190510	89	20200510	29	20180505	59	20190524	89	20200518
30	20180521	60	20190522	90	20200522	30	20180517	60	20190605	90	20200530

Supplementary Table 3: Acquisition dates of the Sentinel-1 SLCs used in this study. The SLC images used as reference for the coregistration are in bold.
